# Assessing chronic effects of chemical pollution on biodiversity using mean species abundance relationships

**DOI:** 10.1093/etojnl/vgaf015

**Published:** 2025-01-16

**Authors:** Venja S A M Schoenke, Jiaqi Wang, Paul J Van den Brink, A Jan Hendriks

**Affiliations:** Department of Environmental Science, Radboud University Nijmegen, Nijmegen, Gelderland, The Netherlands; Department of Environmental Science, Radboud University Nijmegen, Nijmegen, Gelderland, The Netherlands; Aquatic Ecology and Water quality Management Group, Wageningen, Gelderland, The Netherlands; Department of Environmental Science, Radboud University Nijmegen, Nijmegen, Gelderland, The Netherlands

**Keywords:** species sensitivity distribution, potentially affected fraction, long-term assessment, predictive modeling

## Abstract

Because chemical pollution poses a persistent threat to freshwater ecosystems and biodiversity, innovative methodologies are required to address the ecological risks associated with such pollutants. This study predicts the long-term impacts of chemicals based on an equation that describes the time dependency of the median lethal and effect concentration (L(E)C50) with the critical body residue concept. This way, the methodology can predict species sensitivity distributions for any given time point. The methodology was extended to predict the mean species abundance relationships (MSAR) as an indicator of biodiversity. To test and validate the methodology, data from a case study with six freshwater arthropods exposed short- and long-term to imidacloprid were used. The potentially affected fraction of species (PAF) and its opposite (1-PAF) were used to validate the MSAR framework itself. The accuracy of the predicted chronic LC50 values was species-dependent. However, except for one species, all predicted chronic LC50 values were within the 95% confidence intervals (CIs) of the fits based on only acute data. The mean differences between the predicted and calculated MSARs were between 2% and 6%. The predicted MSARs generally underestimated the impact of imidacloprid. However, all predicted MSARs were either similar or lower than the calculated 1-PAF, and their CIs covered the calculated MSARs. Thus, the study found that the presented methodology is useful for predicting the long-term effects of chemical pollutants.

## Teaser

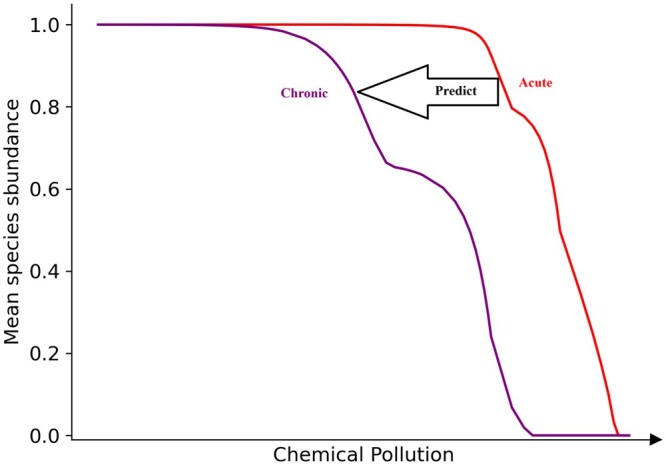

## Introduction

Chemical pollution is posing a growing challenge to biodiversity due to its long-term impact on our natural environment. Addressing the ecological risks associated with such pollutants requires innovative methodologies to understand and overcome these challenges better (Groh et al., 2022; Harrison, 2001; Malaj et al., 2014; Sigmund et al., 2023; Sylvester et al., 2023).

Even though the risk of long-term chemical exposure has been known for years, acute data are more commonly reported than chronic because acute tests take fewer resources and laboratory analyses (U.S. Environmental Protection Agency, 1994). However, chronic exposure situations are more likely to occur in the field (Calow & Forbes, 2003; Hermann et al., 2023). Therefore, it is common practice to use predictive tools to estimate the chronic effects of a substance based on the existing acute data (Eitzer et al., 1996).

The median lethal concentration (LC50) is one of the commonly derived values of toxicity tests (U. S. Environmental Protection Agency, 1994). The predictions in this context rely on the time dependency of these the median lethal and effect concentration (L(E)C50) values. This means that the L(E)C50 values vary depending on when they are measured, because toxicity is also time-dependent (Boyd, 2005). The time dependency of L(E)C50 values was theoretically derived in Hendriks (1995) from the critical body residue concept (McCarty, 1986; McCarty & Mackay, 1993). This methodology can be used to evaluate chronic impacts on species sensitivity distributions (SSDs; Posthuma et al., 2001) to extrapolate biodiversity metrics such as species richness (Wang et al., 2021, 2023).

Because methods that explicitly consider changes in species abundance over time are needed to assess the ecological impact of chemical pollution, in the present study the methodology was extended to derive the change in mean species abundance (MSA;EU, 2020). The mean species abundance relationship (MSAR) has been developed by Hoeks et al. (2020) to assess biodiversity dependent on chemical concentration in response to the need to comprehensively assess the ecological risks posed by chemical pollutants. Mean species abundance relationship connects the concentration of a chemical pollutant in the water to the number of species in the ecosystem by examining the exposure-response relationships of each species to the pollutant, considering both their chances of survival and their capacity to reproduce (Hoeks et al., 2020).

So far, this approach only applies to a specific time point, and the applicability of MSAR for predictive purposes and chronic effects is largely unknown. By predicting MSARs for chronic levels, the present research seeks to provide a clearer perspective on the long-term direct impacts of these pollutants on the overall species count in these ecosystems. This enhanced understanding can lead to better environmental conservation and risk assessment.

Consequently, the present study aimed to predict chronic MSARs for assessing the long-term ecological effects of chemical pollution on biodiversity. The methodology was tested on published acute and chronic data on the impact of imidacloprid on multiple freshwater arthropods and validated on the chronic data that were not used for parametrization (Roessink et al., 2013). Additionally, the MSARs were compared to the potentially unaffected fraction (1-PAF) to validate the MSAR framework. Although the MSAR represents a promising new concept, additional work is required to establish its reliability and utility in regulatory contexts. This comparison was conducted because the 1-PAF is an established and widely accepted metric in these settings. The 1-PAF was derived from the SSD based on the calculated chronic LC50 data (Posthuma et al., 2001). Because data for multiple chronic time points was available, two variants for the prediction were presented. The first variant used only acute data to predict chronic L(E)C50 values, whereas the second variant incorporated the available chronic data up to the predicted time point. This aimed to determine the importance of chronic data in assessing risks.

## Material and methods

### Theory

The calculation of the MSAR is based on exposure-response relationships for survival and reproduction, which can be represented similarly as in Hoeks et al. (2020):


Equation 1 $$\frac{y\left(C\right)}{1-b}=\frac{1}{1+{\left(\frac{C}{L\left(E\right)C50}\right)}^{n_{H}}}$$

where $y\left(C\right)$ represents the fraction survival dependent on concentration (C), L(E)C50 is the median lethal or effect concentration, and $n_{H}$ is the Hill slope, which represents the steepest part of the curve (Gadagkar & Call, 2015). The background mortality b in the fraction was implemented according to (Roessink et al., 2013). If reproduction data are available, the EC50 should be obtained for multiple time points to increase the accuracy of the MSAR.

To calculate the MSAR for different time points, the LC50 and EC50 values are considered time-dependent. To predict the L(E)C50 values over time, Equation 2 in Hendriks (1995) was applied:


Equation 2 $$L\left(E\right)C50\left(t\right)=\frac{L\left(E\right)C50\left(\infty \right)}{1-e^{-k*t}}$$

The resulting equation now represents L(E)C50 values over time (t), with elimination rate constant k, and gives the parameter L(E)C50(∞) as the median lethal or effect concentration at infinity.

The Hill slope values are assumed to be constant over time and are calculated by taking the weighted average. The MSAR is then derived with the calculated Hill slope value and L(E)C50 values of a chosen time point. A case study is presented to validate the methodology, where the predicted chronic MSAR is compared to a calculated chronic MSAR.

### Case study

#### Data collection and treatment

To test the model, we used the data from Roessink et al. (2013), which evaluated imidacloprid’s acute and chronic toxicity to a range of freshwater arthropods (P.J. Van den Brink, personal communication, April 20, 2023). These data were chosen because acute and chronic data were available from the same source under similar testing conditions. Only the species with available acute and chronic data were considered in the current analysis. Table 1 shows the testing conditions of the chosen species. For every treatment, three replicates were conducted in each of the experimental tests. After Day 4 of the acute tests, the individuals were removed from the medium and set to clean water, and observations were done on Days 7 and 9. The mean cumulative mortality was calculated for all scenarios from the control to retrieve the background mortality. However, for data fitting, we used the corresponding survival probabilities.

**Table 1.** Testing conditions, which included a control for the species, and each test had three replicates, with 10 individuals per replicate.

Species	Acute tests		Chronic tests	
	Test concentration (μg/L)	Day(s)	Test concentration (μg/L)	Day(s)
Macrocrustaceans:				
*A. aquaticus*	10, 30, 100, 300, 1,000	0, 1, 2, 4, 7, 9	1, 3, 10, 30, 100	0, 7, 14, 21, 28
*G. pulex*	10, 30, 100, 300, 1,000	0, 1, 2, 4, 7, 9	1, 3, 10, 30, 100	0, 7, 14, 21, 28
Insects:				
*C. obscuripes*	1, 10, 30, 100, 300	0, 1, 2, 4, 7, 9	0.3, 1, 3, 10, 30	0, 7, 14, 21, 28
*P. minutissima*	1, 10, 30, 100, 300	0, 1, 2, 3, 4, 7, 9	0.3, 1, 3, 10, 30	0, 7, 14, 21, 28
*C. dipterum*	1, 10, 30, 100, 300	0, 1, 2, 3, 4, 7, 9	0.03, 0.1, 0.3, 1, 3	0, 7, 14, 21, 28
*C. horaria*	1, 10, 30, 100, 300	0, 1, 2, 3, 4, 7, 9	0.01, 0.03, 0.1, 0.3, 1	0, 7, 14, 21, 28

#### Hill fit

The data were fitted for each species individually to Equation 1 by using the curve_fit() method from the scipy.optimize module in Python based on the Levenberg-Marquardt algorithm for nonlinear regression (Moré, 1978). Initial parameters for L(E)C50 and the Hill slope $n_{H}$ were estimated to ensure an efficient fit.

Because of the definition of L(E)C50, the mean of the two concentrations between which the survival or immobility probability dropped under 50% was chosen as the starting point. If the survival or immobility probability did not drop below 50% during the testing period, the highest concentration tested was multiplied by 10 to establish a starting point. This decision was based on previous fits without starting values. For the start value of $n_{H}$, Equation 1 was solved with the given data and the estimated start value of L(E)C50. The resulting maximum and minimum values for $n_{H}$ were then used as a range to perform the fitting. Within this range, 10 different start values for $n_{H}\;$were used for the fit, and the best-performing fit values in terms of the standard deviation errors (SE) were used for further calculations.

The SE of each parameter was then applied to compute confidence intervals (CI) by adjusting the parameter values in the formula according to their respective SEs. The coefficient of determination ($R^{2}$) was also calculated using the residuals obtained from the fitting (Cameron & Windmeijer, 1997). If no fit was found (e.g., due to no observed death), the L(E)C50 and $n_{H}\;$values from that specific time point were considered unsuitable for subsequent calculations. Fits with higher SEs than the corresponding L(E)C50 values were discarded as not useful for further calculations (see online supplementary material Tables A4–A7). This same methodology was applied to both acute and chronic exposure scenarios.

#### L(E)C50 fit

Based on L(E)C50 values obtained for multiple time points, Equation 2 was fitted with the same algorithm as Equation 1. The SE of the L(E)C50 values were included in the fitting by the sigma function of curve_fit. The bounds of the parameters were set to non-negative values, and L(E)C50(∞) was not higher than the calculated L(E)C50 of the latest known time point. To examine if parametrization on acute data is good enough to predict chronic exposure or if chronic data are needed to obtain a decent prediction, two options were tested. In the “acute-only” option, only acute L(E)C50 values were fitted to Equation 2. The “acute + chronic” option allowed the algorithm to fit Equation 2 also to chronic L(E)C50 values up to but not including the time to be predicted. So, for the prediction of Day 28, the L(E)C50 values of Days 7, 14, and 21 could be used for the fit (Table 1).

#### MSAR calculation

The MSAR was calculated as described in Hoeks et al. (2020) briefly summarized below. The change in lifetime fecundity between an exposed population ($R_{0}\left(C\right)$) and their nonexposed equivalents ($R_{0}\left(0\right)$) is defined by Hendriks et al. (2005) as a logistic exposure-response function:


Equation 3 $$\frac{R_{0}\left(C\right)}{R_{0}\left(0\right)}=\frac{1}{1+{\left(\frac{C}{\mathrm{EC}50}\right)}^{n_{H_{1}}}}*\frac{1}{1+{\left(\frac{C}{\mathrm{LC}50}\right)}^{n_{H_{2}}}}$$

with C as concentration, $n_{H1}$ is the corresponding Hill slope of the EC50 values, and $n_{H2}$ is the corresponding Hill slope of the LC50 values. The lifetime fecundity is then translated to a relative decrease in the carrying capacity. Assuming that the population carrying capacity sets the theoretical maximum population size (Van Gils et al., 2004) and is therefore equal to the optimal abundance of a species, Hoeks et al. (2020) uses $\frac{K\left(C\right)}{K\left(0\right)}$ ratios directly for determining the MSA:


Equation 4 $$\mathrm{MSA}\left(C\right)=\frac{1}{N}\;\sum_{i=1}^{N}\frac{K{\left(C\right)}_{i}}{K{\left(0\right)}_{i}}$$

where $\mathrm{MSA}\left(C\right)$ is the mean abundance of all species at concentration C, expressed as a ratio from 0 to 1, K is the carrying capacity, and N is the number of species included in its derivation.

The MSAR calculations for the desired time points were made with the corresponding $n_{H}$ and predicted L(E)C50 values. In this case, the predicted time points were 28 and 21 days because chronic data for validation were available for these specific time points. To compute the MSAR, it was assumed that the reproductive EC50 are the same as the immobilization EC50 values because no reproductive data were available. This assumption is underpinned by the fact that imidacloprid leads to immobilization and that immobilized individuals cannot contribute to the species abundance by reproduction (Nyman et al., 2013). Additionally, the CIs of the MSAR prediction are based on the CIs of the L(E)C50 predictions as well as the $n_{H}\;$calculations. If no data about the lifetime fecundity ($R_{0}$) are available, it can be assumed, according to Savage et al. (2004), that it is constant. Because we had no such data available, the algorithm was used in the simplified form from Hoeks et al. (2020) with a lifetime fecundity of $R_{0}(0)=4$. For the validation of the prediction, the chronic data from Roessink et al. (2013) were fitted to Equation 1, and the values of the L(E)C50 and $n_{H}\;$were used to calculate the MSAR.

#### PAF calculation

The SSD serves as a statistical framework modeling the sensitivities of various species to a given toxic compound by providing the PAF over the concentration of the compound data (Posthuma & de Zwart, 2012; Posthuma et al., 2001). The PAF in this study is calculated with the chronic LC50 values obtained from the fit of Equation 1. The Excel SSD Generator provided by the U. S. Environmental Protection Agency was used to calculate the SSD (U. S. Environmental Protection Agency, 2016). The Excel SSD Generator uses a normal distribution to convert proportions into probit values, ensuring that all values remain non-negative by centering the distribution with a mean of 5 and a standard deviation of 1. The arithmetic mean was used to calculate central tendencies. The resulting PAF values were then translated in the 1-PAF to provide a visual comparison with the MSAR.

## Results

To analyze the quality of the model, we examined the results of each step with qualitative and quantitative criteria.

### Hill slope

The fits of Equation 1 resulted in at least three time points from the acute data that were deemed suitable for further calculations. The L(E)C50 and slope ($n_{H}$) values of the acute and chronic exposure differed from those reported in Roessink et al. (2013) because a different fitting algorithm was used. However, the fitted L(E)C50 values were within the CIs reported in Roessink et al. (2013), indicating satisfactory fits (see online supplementary material Tables A1 and A2).

**Table 2.** The $R^{2}$ values for fitting Equation 2 to calculated median lethal concentration values.

Species	Acute-only	Day 21 acute + chronic	Day 28 acute + chronic
*A. aquaticus*	0.56	0.63	0.69
*C. horaria*	0.79	0.82	0.28
*C. obscuripes*	0.93	0.13	0.22
*C. dipterum*	0.15	−0.25	−0.35
*G. pulex*	0.99	0.99	0.94
*P. minutissima*	0.99	0.91	0.92

### L(E)C50

**Figure 1.**
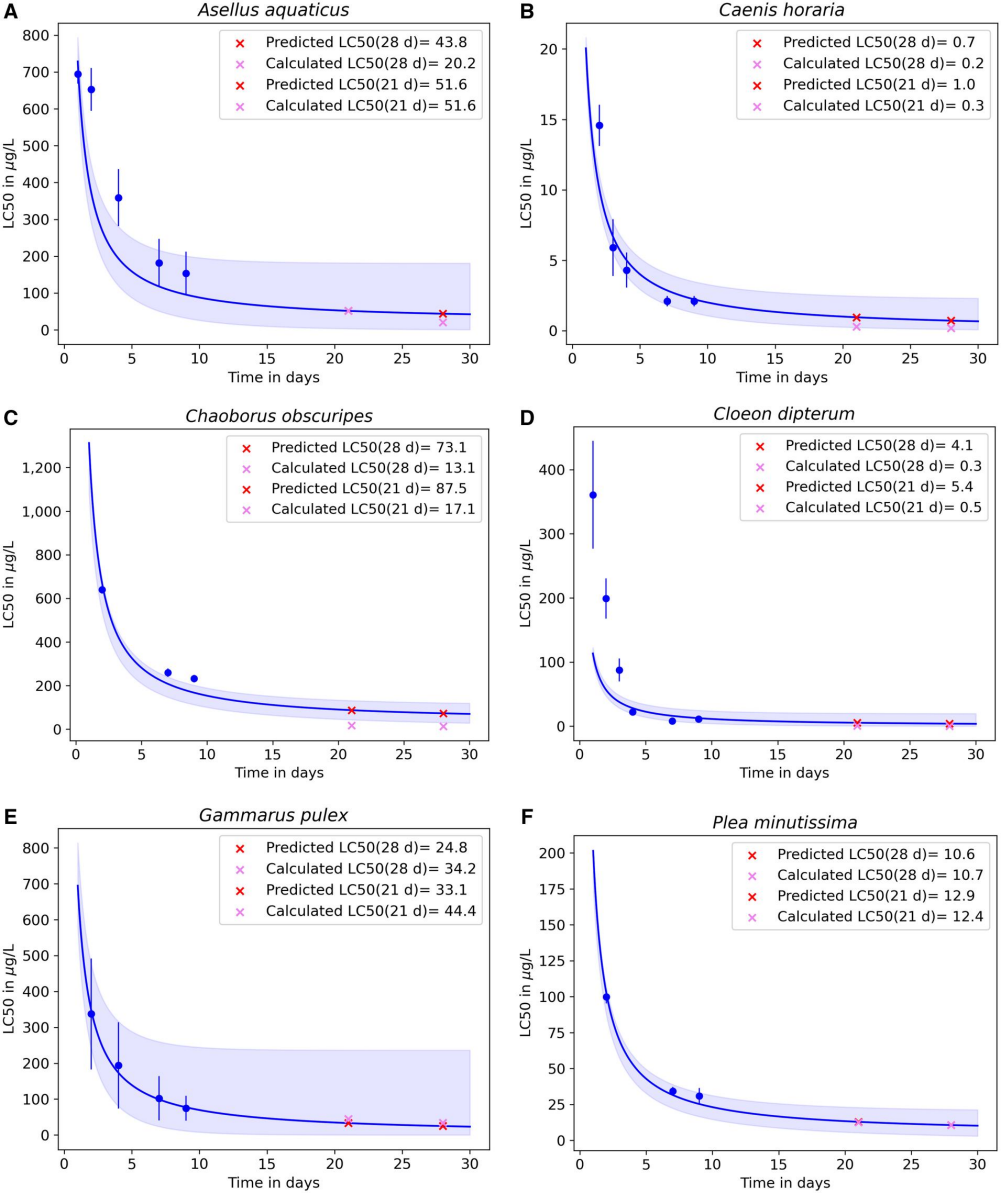
Median lethal concentrations (LC50) as a function of time t (blue curve) with confidence intervals (blue shade) by fitting Equation 2 to acute data of all six species. Data represent the calculated LC50 values (blue dots) with standard errors (blue bars). The predicted LC50 values (red crosses) and the calculated LC50 values (pink crosses) at Days 21 and 28 are shown in the legend.

For the fits of Equation 2, Figure 1 shows the results of the fits based only on acute LC50 values for all species (see online supplementary material Tables A8 and A9 for parameter values). The calculated LC50 values for Days 21 and 28 are within the CI of the fits for all species except *Chaoborus obscuripes*. The fit for *C. obscuripes* has an *R*^2^ value of 0.93, but only three data points were available for the fit. When chronic data points were taken into account, the *R*^2^ values dropped for this species (Table 2). *Asellus aquaticus* and *Cloeon dipterum* have the lowest *R*^2^ values (Table 2) and show an underestimation of the chronic LC50 values (Figure 1). For *C. dipterum*, the fits based on acute and chronic data even resulted in negative *R*^2^ values. This can happen when applying *R*^2^ for nonlinear models, and the fit is worse than the baseline model, which predicts the mean of the observed data (Cameron & Windmeijer, 1997). The best fits were obtained for *Gammarus pulex* and *Plea minutissima* with *R*^2^ values over 0.90 for all cases (Table 2).

### MSAR and 1-PAF of species

**Figure 2.**
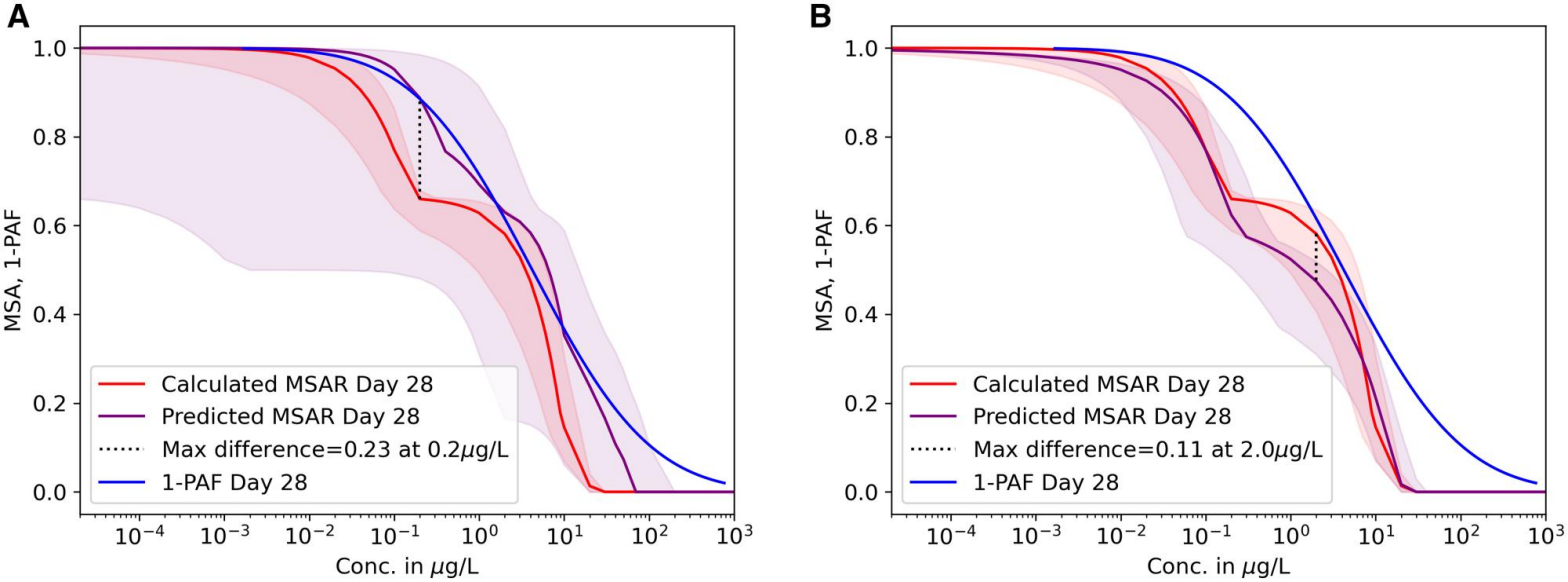
Predicted (violet) and calculated (red) mean species abundance relationships (MSAR) for Day 28 based on only acute data (left) and acute data as well as chronic up to Day 21 (right). Confidence intervals are indicated in shades of the representative color. The concentration is in a log10 scale. The Y-axis shows abundance in fractions.

**Figure 3.**
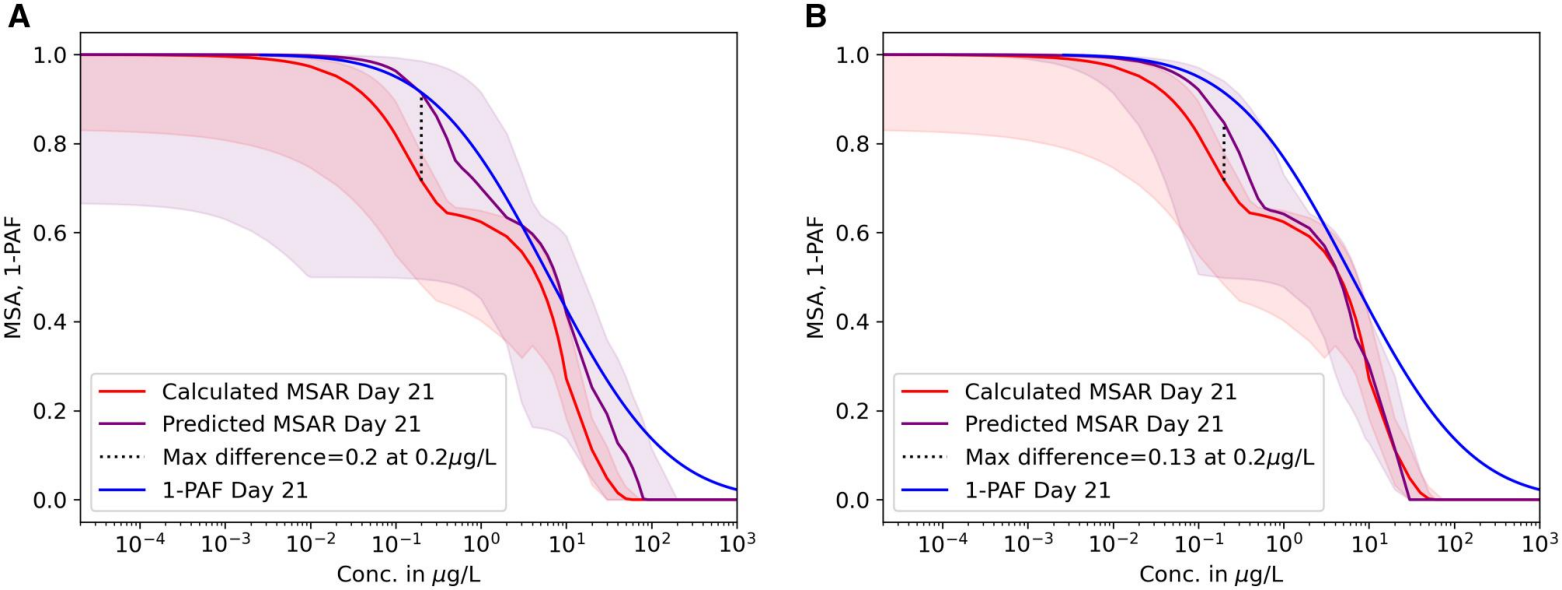
Predicted (violet) and calculated (red) mean species abundance relationships (MSAR) for Day 21 based on only acute data (left) and acute data as well as chronic up to Day 14 (right). Confidence intervals are indicated in shades of the representative color. The concentration is in a log10 scale. The Y-axis shows abundance in fractions.

The predicted and calculated chronic MSA and the 1-PAF over concentration are shown in Figures 2 and 3. In Figure 2, the MSAR was predicted for Day 28 using acute (left) or acute and chronic data available for the fitting (right). If only acute data were used, the predicted MSAR (violet curve) underestimated the effect of imidacloprid when comparing it to the calculated chronic MSAR. However, the CIs of the prediction fully cover the fitted MSAR, and the *R*^2^ value is 0.95 (Table 3). The maximum difference in predicted versus fitted abundance is 0.23 at 0.2 µg/L, and the mean difference 0.06 (Table 3). The 1-PAF shows a similar curve to the predicted MSAR. If chronic data were also included for fitting, the largest difference occurred when the concentration was at 2 μg/L, where the predicted curve is lower than the fitted curve. The maximum difference here is 0.11, and the mean difference 0.03. In this case, the 1-PAF curve lays mostly above the predicted and calculated MSAR. For a nearer future (21 days), results are similar (Figure 3). Both predictions show the same pattern as the fitted curve and have high *R*^2^ values (Table 3), while the prediction based on acute and chronic data (Figure 3 right) is slightly better. The 1-PAF curves also show the same behavior as in Figure 2. The maximum difference in fraction abundance loss when only predicting based on acute data is 0.2 at 0.2 μg/L and lowers to 0.13 when considering chronic data (Table 3). The mean difference using only acute data was 0.05 and using also chronic data 0.02. The largest difference between the prediction and the fitted MSAR can be seen for lower concentrations at the beginning of the decline in MSA. The underestimation at the beginning of the decline, as well as the rest of the calculated MSARs, are still covered by the CIs.

**Table 3.** The $R^{2}$, maximum difference, and mean difference values between the predicted and calculated mean species abundance relationships.

	$R^{2}$	Maximum difference	Mean difference
Day 21 acute-only	0.96	0.2	0.05
Day 21 acute + chronic	0.99	0.13	0.02
Day 28 acute-only	0.95	0.23	0.06
Day 28 acute + chronic	0.99	0.11	0.03

## Discussion

We have demonstrated that Equation 2 predicting chronic LC50 values was mostly accurate for the species tested. Yet, the fit was inadequate for certain species such as *C. dipterum*. We used the predicted LC50 values to calculate MSARs and the 1-PAF at various time points. These calculations were then validated with chronic data.

### Interpretation of results

For the L(E)C50 fits, acute data from Days 7 and 9 were used. Because the species had been transferred to clean water on Day 4, recovery processes can explain low *R*^2^ values and qualitative misfits. The LC50 fits for *C. obscuries*, *C. dipterum*, and *P. minutissima* were most affected. In the case of *C. obscuries*, this effect cannot be seen in Figure 1 because only three LC50 values were used for this fit, whereas two of them were from Days 7 and 9. However, if chronic data were considered (see online supplementary material Figure A1), the *R*^2^ values dropped from 0.93 to 0.13 and 0.22 because the LC50 values for Days 7 and 9 are outliers in these fits. The same effect occurred for *P. minutissima*, but for this species, recovery seems slower than for *C. obscuries* (see online supplementary material Figure A1). For *C. dipterum*, the effect is not as strong, but the LC50 value of Day 9 is higher than that of Day 7. The weighted fit can explain the low *R*^2^ values in this case because the SEs of the LC50 values for the first three days are higher than for the later days. The effect of the weighted fit also applies to *A. aquaticus* because, here, the LC50 value of Day 1 has the lowest SE.

For the immobilization data, stronger recovery effects were present (see online supplementary material Figures A2 and A3), resulting in low *R*^2^ values across all fits (see online supplementary material Table A3). Nevertheless, excluding data from Days 7 and 9 would limit the ability to generate fits for certain species, such as *P. minutissima*, further reducing the already small number of species available for constructing SSDs. Given that the LC50 data are the more critical source and are less affected by recovery compared to immobilization data, including data from Days 7 and 9 was deemed necessary to maintain the robustness and utility of the fits.

Not surprisingly, the MSAR becomes more precise when also including chronic values for the fitting (Table 3). Because fewer chronic data could be used to fit the 21-day prediction, the improvement of the prediction also declined in comparison to the prediction of Day 28. However, predictions based on acute data alone were able to represent the chronic effect of imidacloprid because the confidence intervals overlap entirely with the calculated curve. Additionally, the predicted and calculated MSARs followed the same sigmoid pattern as the 1-PAF, but the curves for 1-PAF are slightly higher than the fitted MSAR curves on Day 21 and Day 28. In this analysis, the value of R0 was fixed at 4, which is suitable for risk assessment because it represents a relatively low estimate. This ensures a conservative approach to assessing species abundance under stress. If R0 were increased, the performance of the predicted MSAR would improve compared to the calculated MSAR. However, this change would also shift the MSAR curve to the right, making it more similar to the 1-PAF curve (see online supplementary material Figure A4). In a study by Thunnissen et al. (2022), it was demonstrated that the MSAR is a better representation of the relative abundance in the field than the commonly used 1-PAF in environmental quality assessment. The additional calculations required to compute the MSAR are therefore justified in terms of ecological relevance.

The mean and maximum difference did not change from the 21-day prediction to the 28-day prediction when only using acute data. Additionally, the mean and maximum difference improved for Day 28 compared to Day 21 when chronic data were included for the fits. This could indicate that the approach is especially suitable for long-term exposure, which typically occurs in the field (Calow & Forbes, 2003). So, our model facilitates field relevant indicators from acute data.

### Limitations and outlook

First, the assumption of the median immobilization concentration being equal to the EC50 of reproduction could be a limitation. Because the same assumption was made for the calculated MSAR, a similar behavior is expected. However, a comparison of the MSAR and 1-PAF (Thunnissen et al., 2022) has shown that this assumption was reasonable because the curves are similar. The 1-PAF used in this study includes six species instead of the recommended eight for environmental quality standard settings (Europen Food Safety Authoriy Panel on Plant Protection Products and their Residues, 2013). So, the 1-PAF serves primarily as a comparative tool for evaluating the MSAR framework rather than as a standalone regulatory benchmark. Second, the prediction based only on the time dependency of L(E)C50 values does not consider a possible change in modes of action from acute to chronic behavior of imidacloprid (Nauen, 1995). Third, the underestimation of the effect of imidacloprid could be explained by its toxicokinetics. Imidacloprid has a slow uptake and biotransforms to imidacloprid-olefin. Although biotransformation often deactivates substances, activation to more toxic compounds may also cause deviations (Macherey & Dansette, 2008). Imidacloprid-olefin is also toxic and is hypothesized to bind irreversibly with the receptor (Huang et al., 2021). The irreversible binding causes a steady increase in the internal body burden over time, because the chemical continues to bind to receptors. This means that even at very low concentrations, prolonged exposure may eventually lead to death. This effect was most noticeable for *C. dipterum*, because both imidacloprid and its biotransformation product, imidacloprid-olefin, were similarly toxic to this species, and almost no elimination of the substances was observed (Huang et al., 2021). As a result, the proposed framework may not fully apply to *C. dipterum* exposed to imidacloprid, because a steady state cannot be achieved. For *G. pulex*, however, the effect was less severe due to its slightly faster elimination rate compared to *C. dipterum* (Huang et al., 2021). Despite these limitations, the overall goal of predicting the effect on a community level, however, was achieved and general information can be given by the presented approach. To gather more information on the robustness of the approach to irreversible binding, thiacloprid could also be evaluated (Raths et al., 2023). Additionally, it would be interesting to compare imidacloprid with another data set, for example, chlorpyrifos, whose uptake is much faster (Rubach et al., 2010).

## Conclusion

In conclusion, our study demonstrates that by using the predicted L(E)C50 values, we can calculate the MSARs at various time points, which we validated using chronic data. The findings indicate that model precision improves when chronic values are included in the fitting process. However, the predictions can anticipate the calculated MSARs based on only acute data when considering the CIs. Additionally, the mean and maximum differences between the calculated and predicted MSAR remained the same or decreased when predicting over longer time scales. The predicted MSARs closely align with the calculated 1-PAF curves, supporting their ecological relevance, as Thunnissen et al. (2022) highlighted. Our method bridges the gap between acute toxicity data and long-term ecological impacts, providing valuable insights for environmental quality assessment.

## Supplementary Material

vgaf015_Supplementary_Data

## Data Availability

The data used in this paper are available in the online supplementary material (Tables A10 and A11). The calculation tools used are openly available in Python.
