# Supplementary material for: Assessing chronic effects of chemical pollution on biodiversity using mean species abundance relationships
Source: Environ Toxicol Chem. 2025 Jan 16;44(4):1134–41. doi: 10.1093/etojnl/vgaf015 (PMC11947378; doi:10.1093/etojnl/vgaf015)
Supplement: vgaf015_Supplementary_Data [file vgaf015_supplementary_data.zip › vgaf015_Supplementary_Data/SI Figures A1-A4.pdf]

Supplemental Data

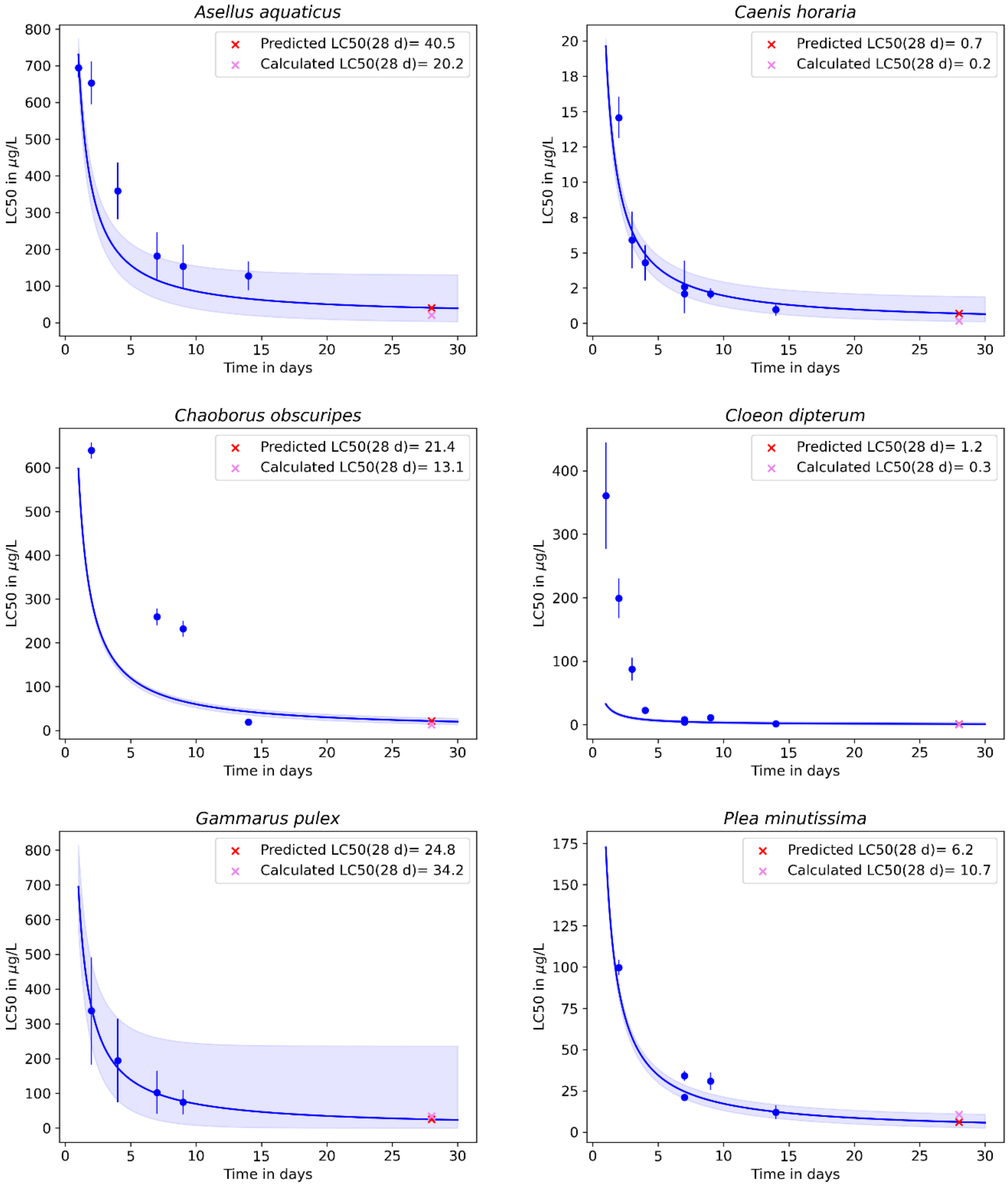

Figure A1: Median lethal concentrations (LC50) as a function of time t (blue curve) with confidence intervals (blue shade) by fitting Equation 2 to acute + chronic data of all six species. Data represents the calculated LC50 values (blue dots) with standard errors (SE) (blue bars). The predicted LC50 value (red cross) and the calculated LC50 value (pink cross) at day 28 is shown in the legend.

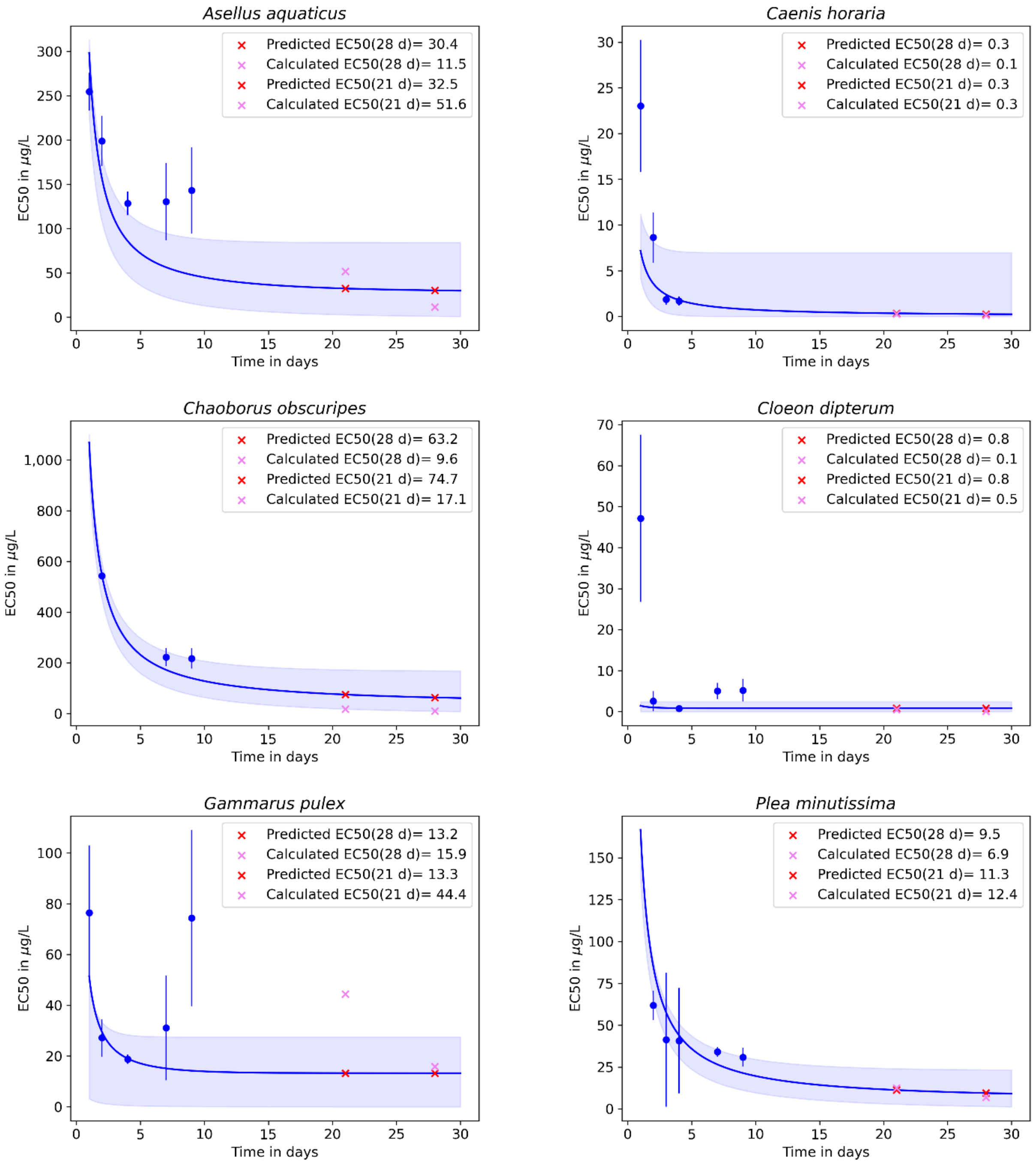

Figure A2: Median effect concentration (EC50) as a function of time t (blue curve) with confidence intervals (blue shade) by fitting Equation 2 to acute data of all six species. Data represents the calculated EC50 values (blue dots) with standard errors (blue bars). The predicted EC50 values (red crosses) and the calculated EC50 values (pink crosses) at day 21 and 28 are shown in the legend.

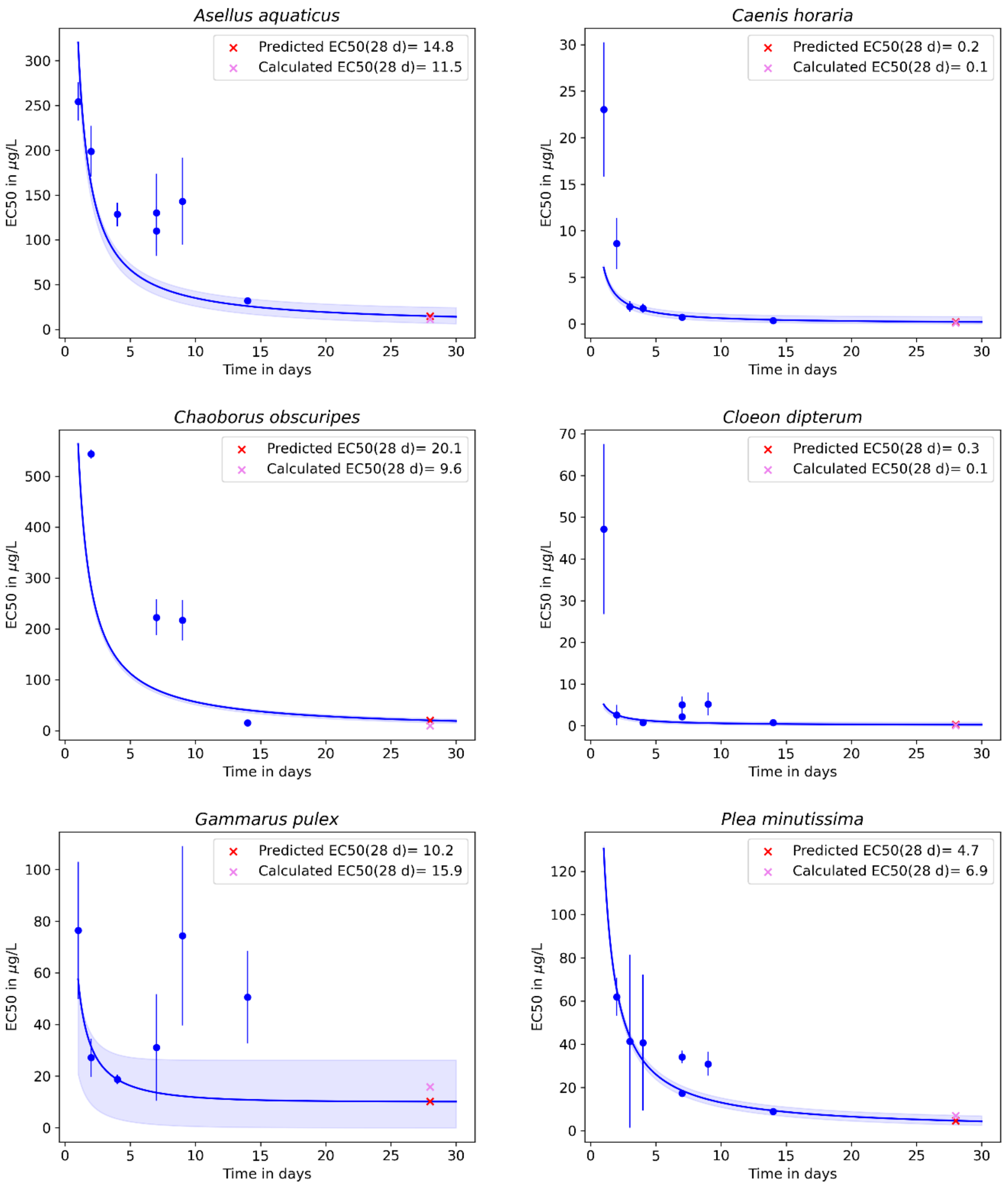

Figure A 3: Median effect concentrations (EC50) as a function of time t (blue curve) with confidence intervals (blue shade) by fitting Equation 2 to acute + chronic data of all six species. Data represents the calculated EC50 values (blue dots) with standard errors (SE) (blue bars). The predicted EC50 value (red cross) and the calculated EC50 value (pink cross) at day 28 is shown in the legend.

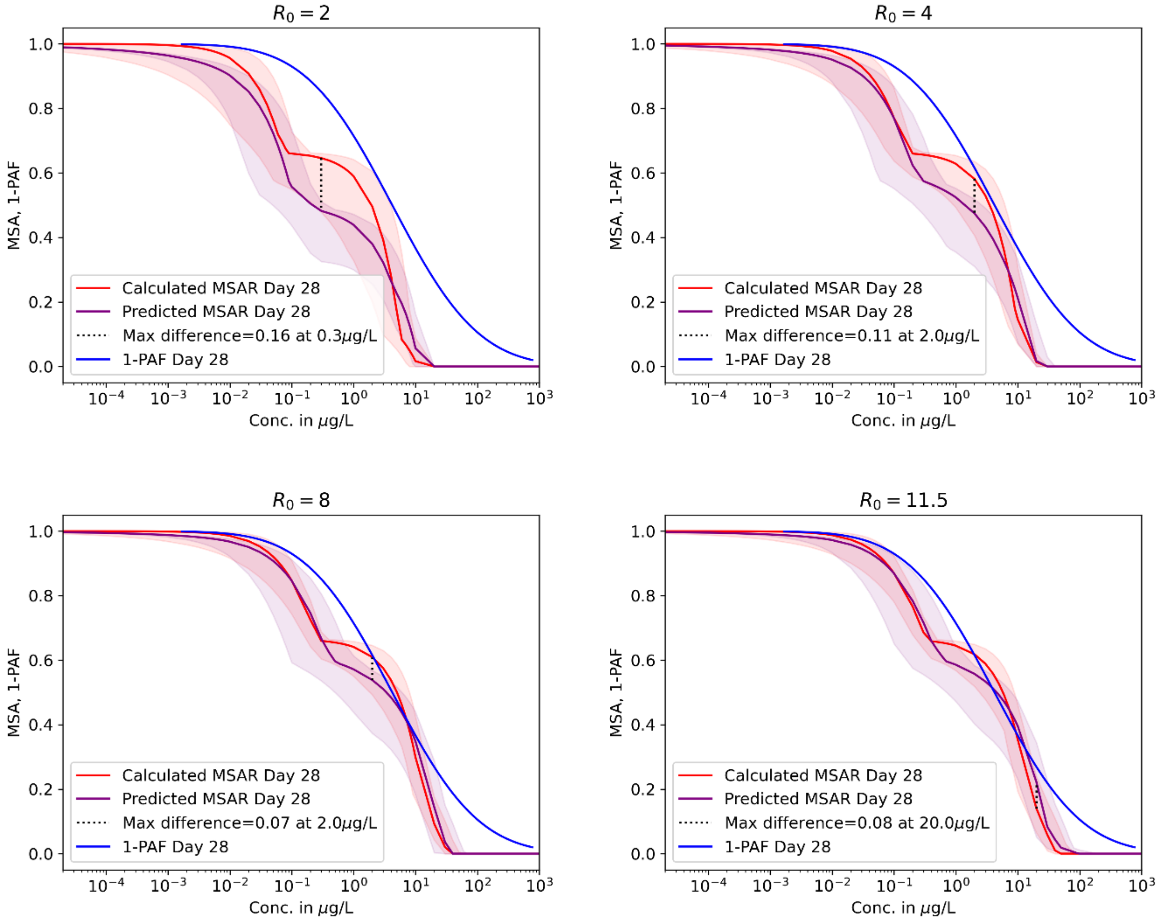

Figure A4: Predicted (violet) and calculated (red) Mean Species Abundance Relationships (MSAR) for day 28 based on acute + chronic data up to day 21 for multiple R_0 values based on the range from 2-11.5 from (Hendriks, 2007). Confidence intervals are indicated in shades of the representative color. The concentration is in a log10-scale. The Y-axis shows abundance in fractions.
