# Supplementary material for: Assessing chronic effects of chemical pollution on biodiversity using mean species abundance relationships
Source: Environ Toxicol Chem. 2025 Jan 16;44(4):1134–41. doi: 10.1093/etojnl/vgaf015 (PMC11947378; doi:10.1093/etojnl/vgaf015)
Supplement: vgaf015_Supplementary_Data [file vgaf015_supplementary_data.zip › vgaf015_Supplementary_Data/Tables SI_A1-A3.docx]

*Table A 1: Fitted median lethal concentration (LC50) values compared to LC50 values and their Confidence intervals from Roessink (2013).*

| Species |  |  |  |
| --- | --- | --- | --- |
| Day 4 | LC50 | CI LC50  Roessink et al. | LC50  Roessink et al. |
| *A. aquaticus* | 359 | 216-461 | 316 |
| *C. horaria* | 4.3 | 4.19-10.6 | 6.68 |
| *C. obscuripes* | NA | 247-350 | 294 |
| *C. dipterum* | 22.5 | 17.7-39.1 | 26.3 |
| *G. pulex* | 194 | 155-446 | 263 |
| *P. minutissima* | NA | NC | 37.5 |
| day 28 |  |  |  |
| *A. aquaticus* | 20.2 | 8.61-47.9 | 20.3 |
| *C. horaria* | 0.18 | NC | 0.316 |
| *C. obscuripes* | 13.1 | 7.33-21.6 | 12.6 |
| *C. dipterum* | 0.317 | 0.113-0.338 | 0.195 |
| *G. pulex* | 34.2 | 20.9-54.6 | 33.8 |
| *P. minutissima* | 10.73 | 7.61-12.6 | 9.8 |
| NC = confidence interval could not be calculated  NA= LC50 values could not be calculated | | | |

*Table A 2: Fitted median effect concentration (EC50) values compared to EC50 values and their Confidence intervals from Roessink (2013).*

| Species |  |  |  |
| --- | --- | --- | --- |
| Day 4 | EC50 | CI EC50  Roessink et al. | EC50  Roessink et al. |
| *A. aquaticus* | 128.57 | NC | 119 |
| *C. horaria* | 1.67 | 1.05–2.99 | 1.77 |
| *C. obscuripes* | NA | NC | 284 |
| *C. dipterum* | 0.74 | 0.460–2.28 | 1.02 |
| *G. pulex* | 18.71 | 8.84–37.8 | 18.3 |
| *P. minutissima* | 40.72 | 31.1–41.5 | 35.9 |
| day 28 |  |  |  |
| *A. aquaticus* | 11.54 | 5.94 – 23.7 | 11.9 |
| *C. horaria* | 0.12 | 0.070 – 0.228 | 0.126 |
| *C. obscuripes* | 9.62 | 8.17 – 17.1 | 11.8 |
| *C. dipterum* | 0.11 | 0.075 – 0.201 | 0.123 |
| *G. pulex* | 15.85 | 9.80 – 24.1 | 15.4 |
| *P. minutissima* | 6.92 | 4.81 – 8.64 | 6.45 |
| NC = confidence interval could not be calculated  NA= EC50 values could not be calculated | | | |

*Table A3: The* $R^{2}$ *values for fitting Equation 2 to calculated median effect concentrations values.*

| Species | Acute-only | Day 21  acute + chronic | Day 28  acute + chronic |
| --- | --- | --- | --- |
| *A. aquaticus* | -0.67 | 0.004 | 0.21 |
| *C. horaria* | 0.08 | 0.18 | -0.02 |
| *C. obscuripes* | 0.88 | 0.21 | 0.29 |
| *C. dipterum* | -0.38 | -0.06 | -0.01 |
| *G. pulex* | -0.48 | -0.99 | -1.04 |
| *P. minutissima* | -0.64 | 0.67 | 0.75 |
