# Supplementary figures and images for: Assessing chronic effects of chemical pollution on biodiversity using mean species abundance relationships
Source: Environ Toxicol Chem. 2025 Jan 16;44(4):1134–41. doi: 10.1093/etojnl/vgaf015 (PMC11947378; doi:10.1093/etojnl/vgaf015)

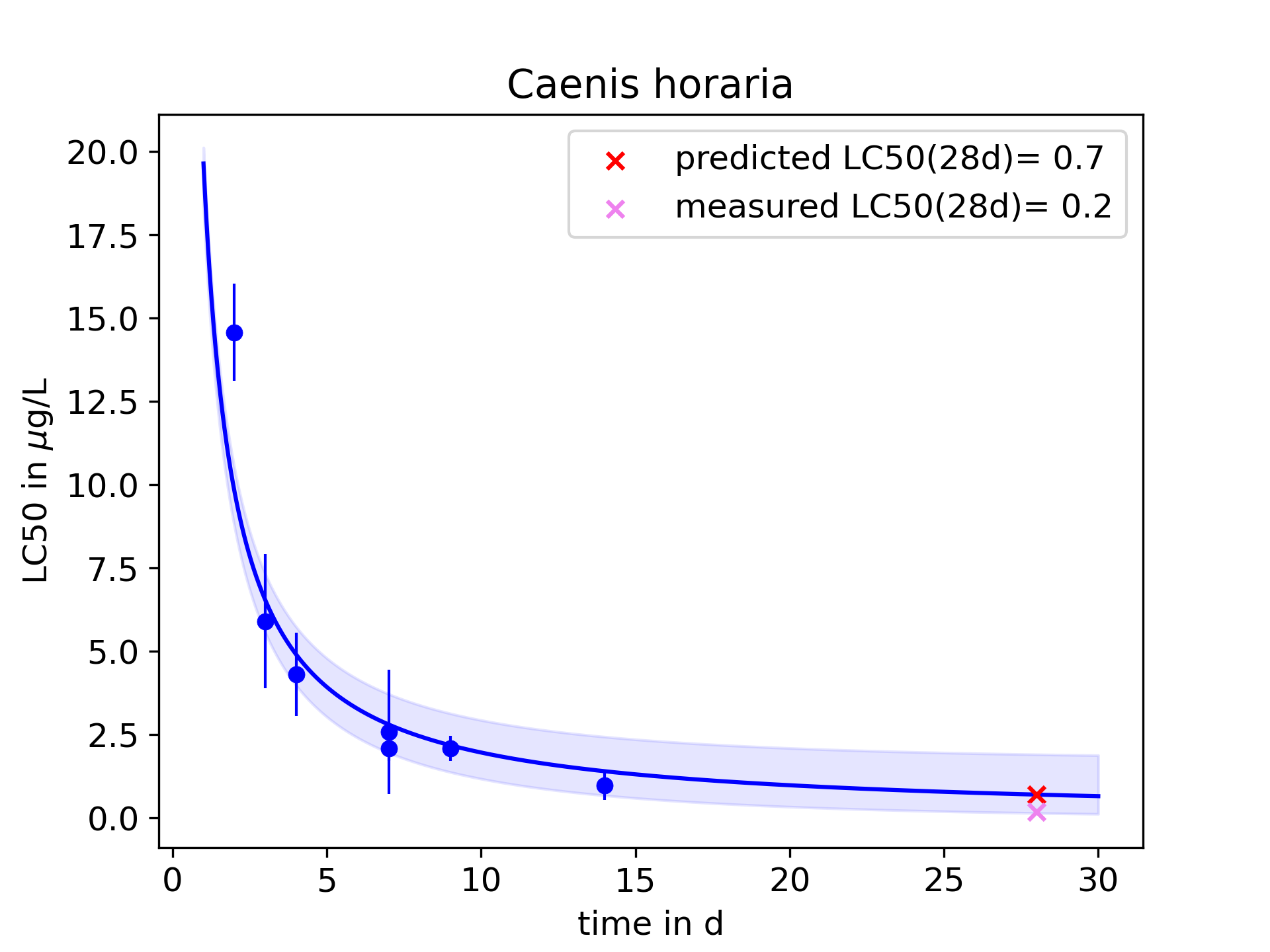

Supplement: vgaf015_Supplementary_Data [file vgaf015_supplementary_data.zip › vgaf015_Supplementary_Data/Figure A1b Caenis horaria LC50fit_with_chronic.tif]

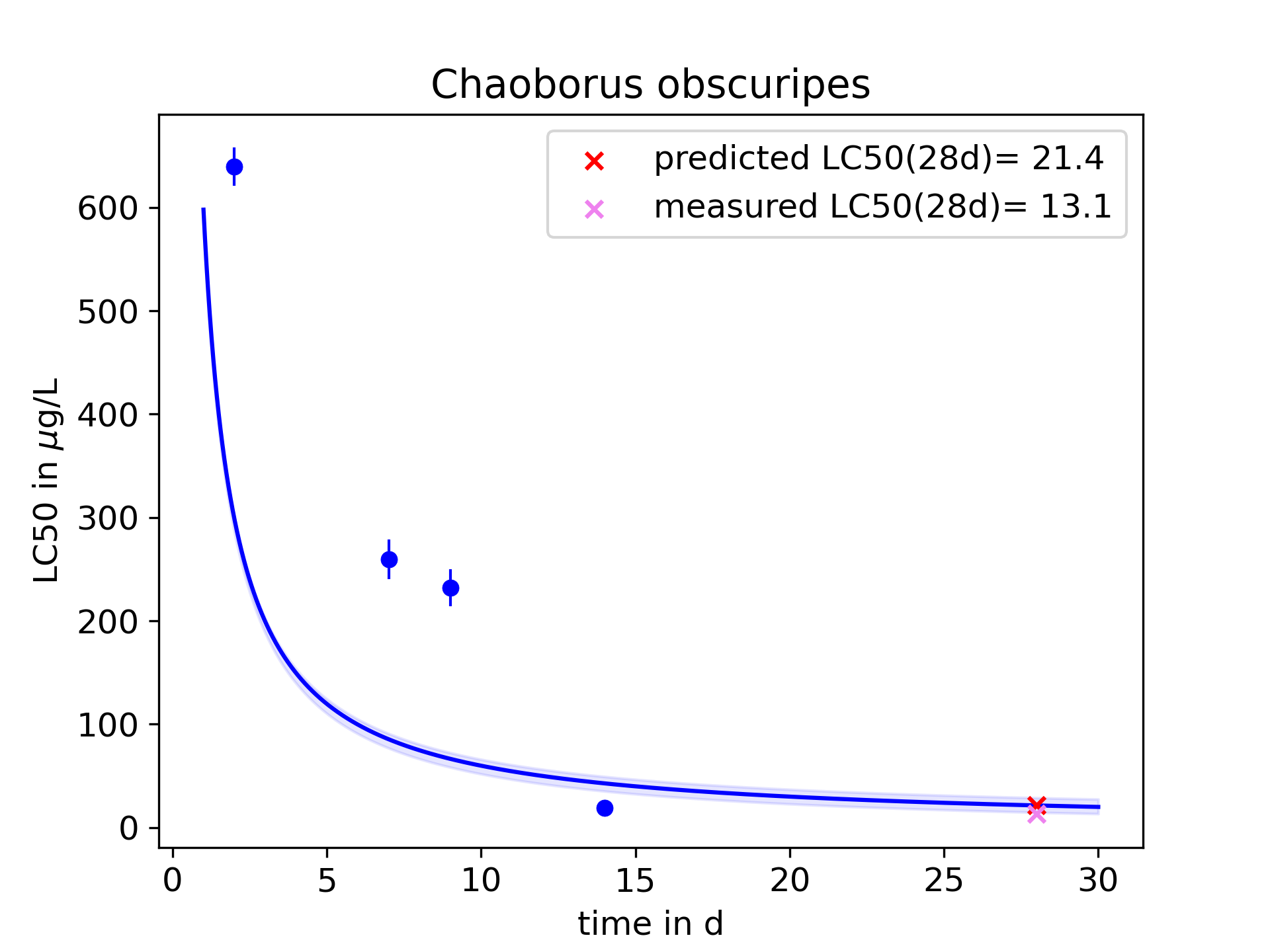

Supplement: vgaf015_Supplementary_Data [file vgaf015_supplementary_data.zip › vgaf015_Supplementary_Data/Figure A1c Chaoborus obscuripes LC50fit_with_chronic.tif]

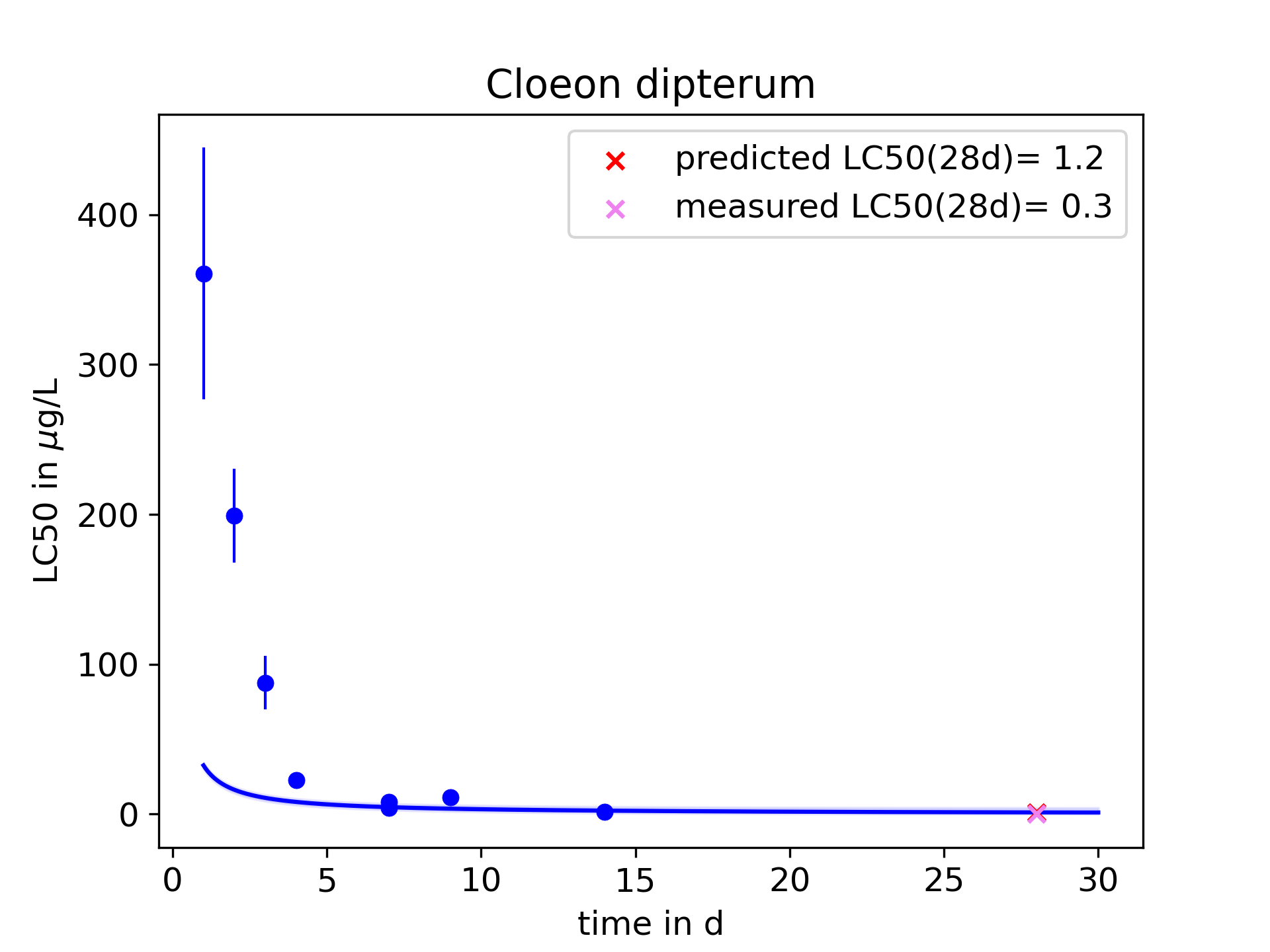

Supplement: vgaf015_Supplementary_Data [file vgaf015_supplementary_data.zip › vgaf015_Supplementary_Data/Figure A1d Cloeon dipterum LC50fit_with_chronic.tif]

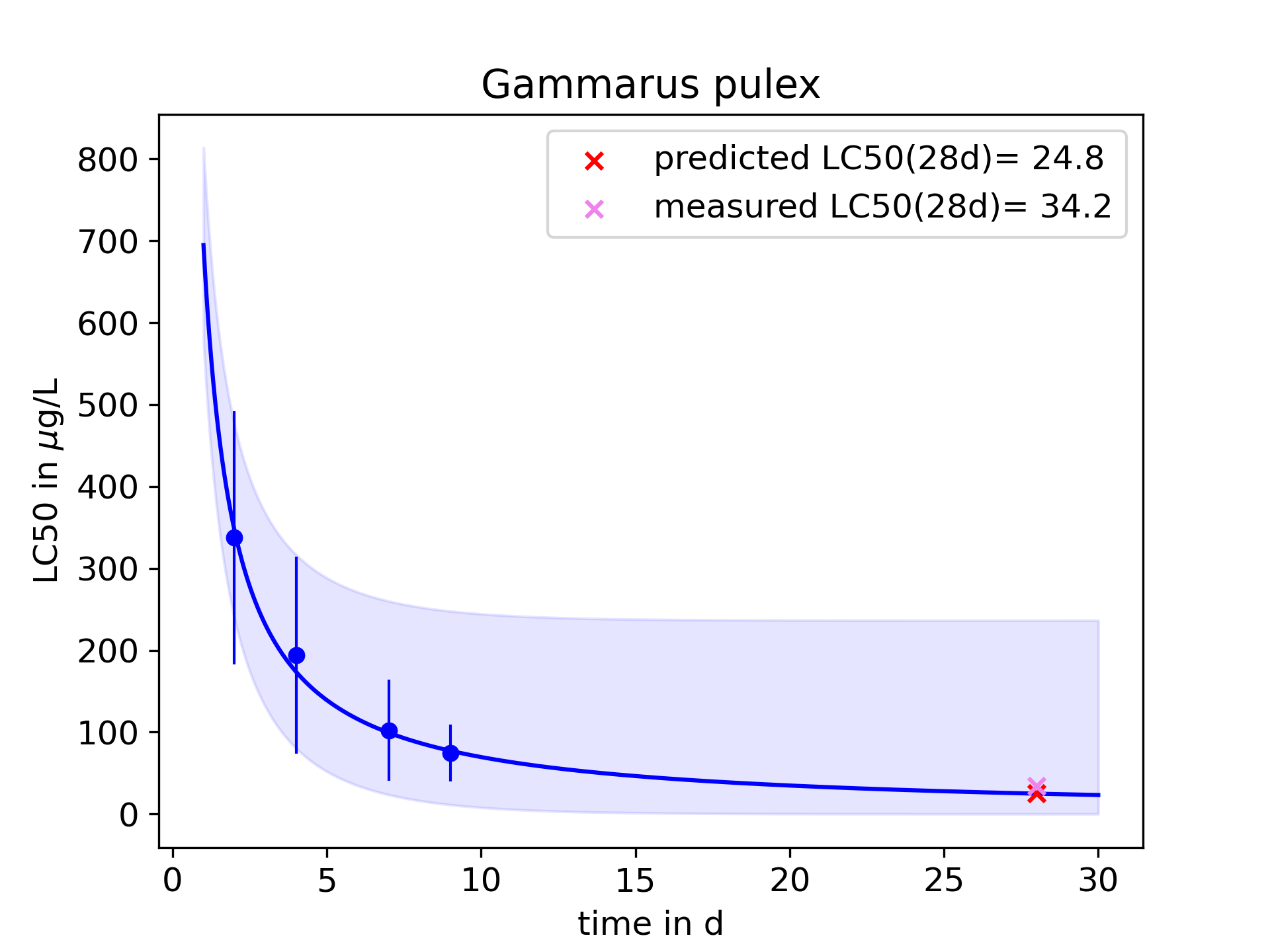

Supplement: vgaf015_Supplementary_Data [file vgaf015_supplementary_data.zip › vgaf015_Supplementary_Data/Figure A1e Gammarus pulex LC50fit_with_chronic.tif]

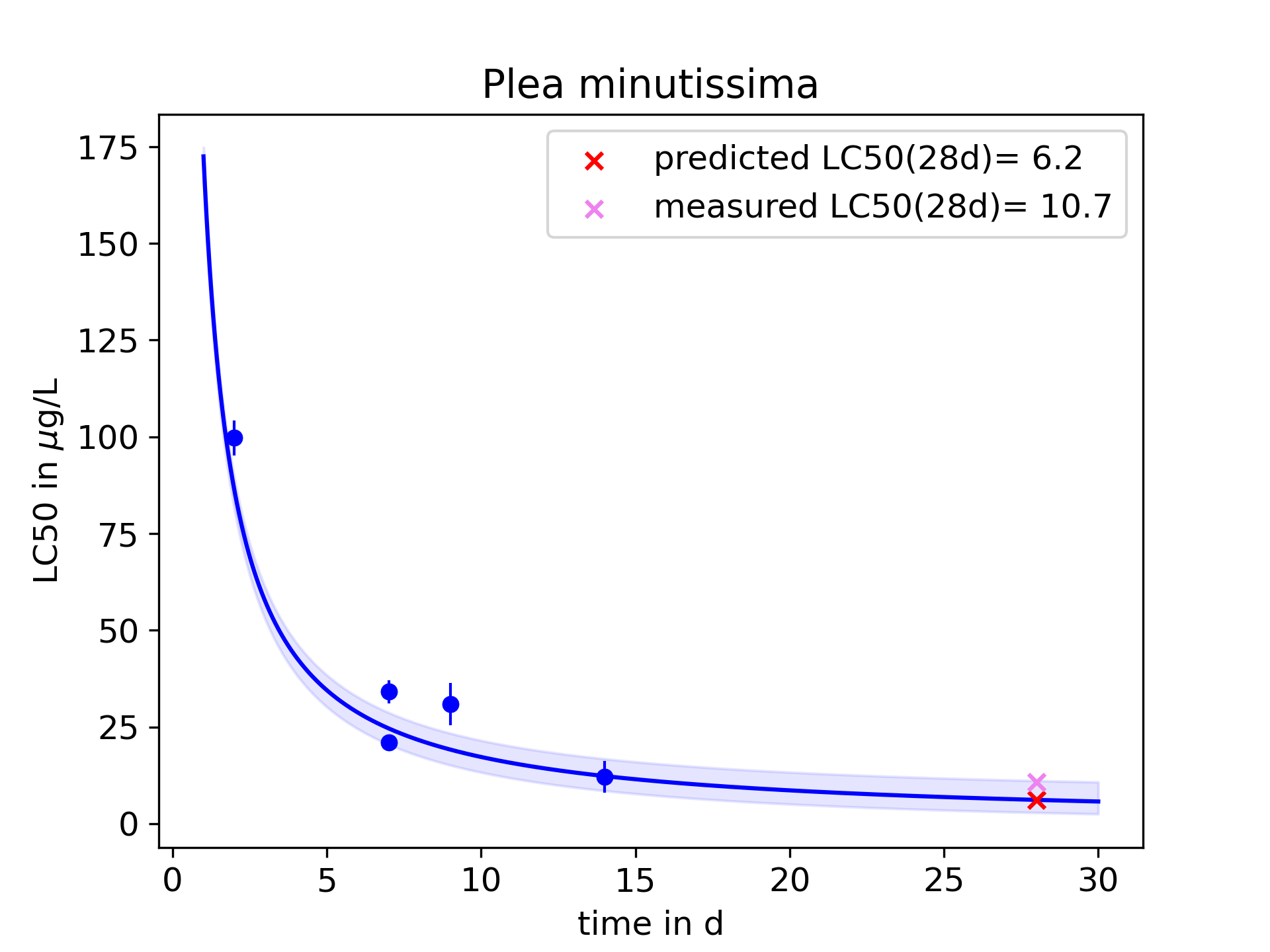

Supplement: vgaf015_Supplementary_Data [file vgaf015_supplementary_data.zip › vgaf015_Supplementary_Data/Figure A1f Plea minutissima LC50fit_with_chronic.tif]

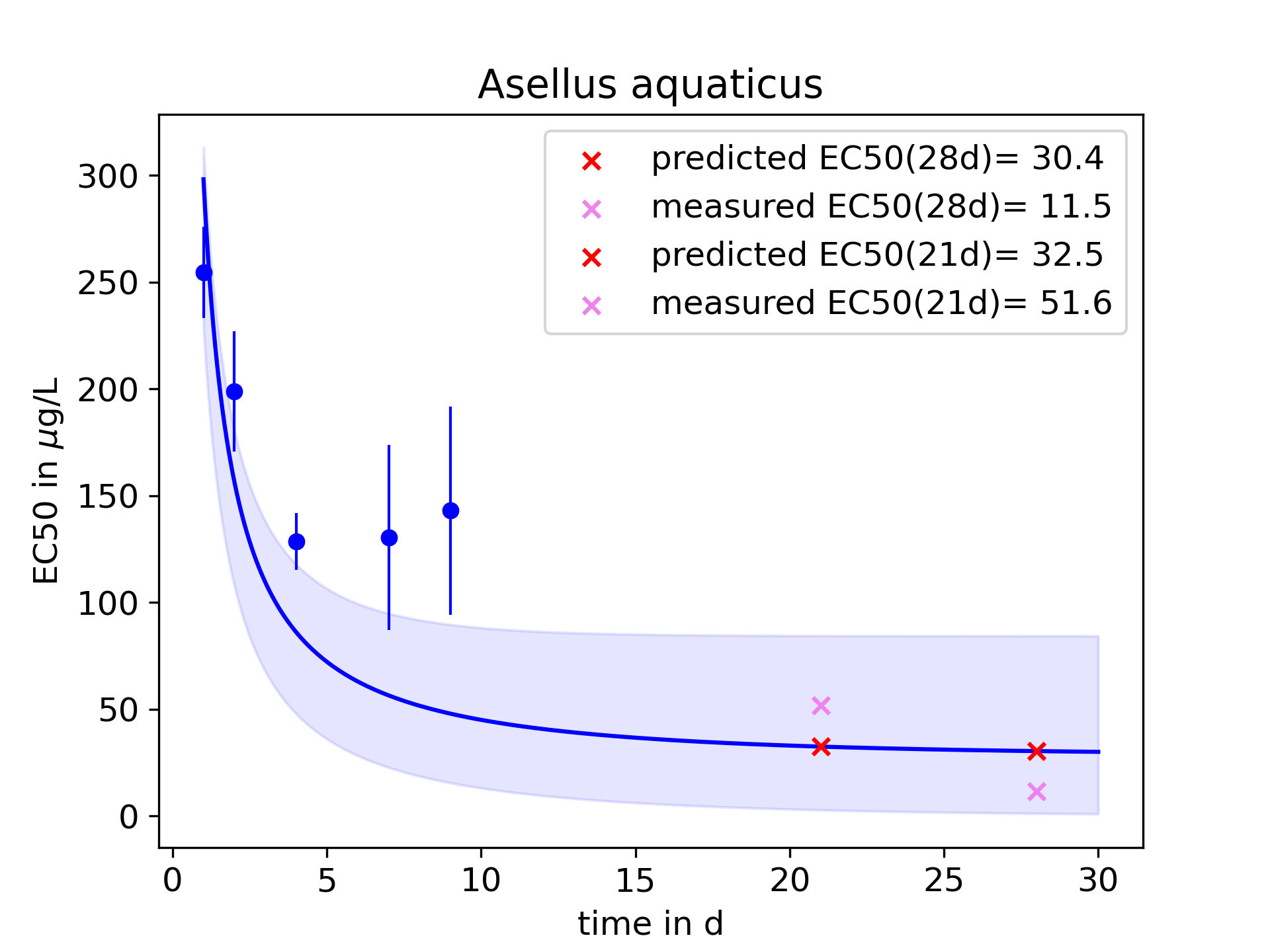

Supplement: vgaf015_Supplementary_Data [file vgaf015_supplementary_data.zip › vgaf015_Supplementary_Data/Figure A2a Asellus aquaticus EC50fit.tif]

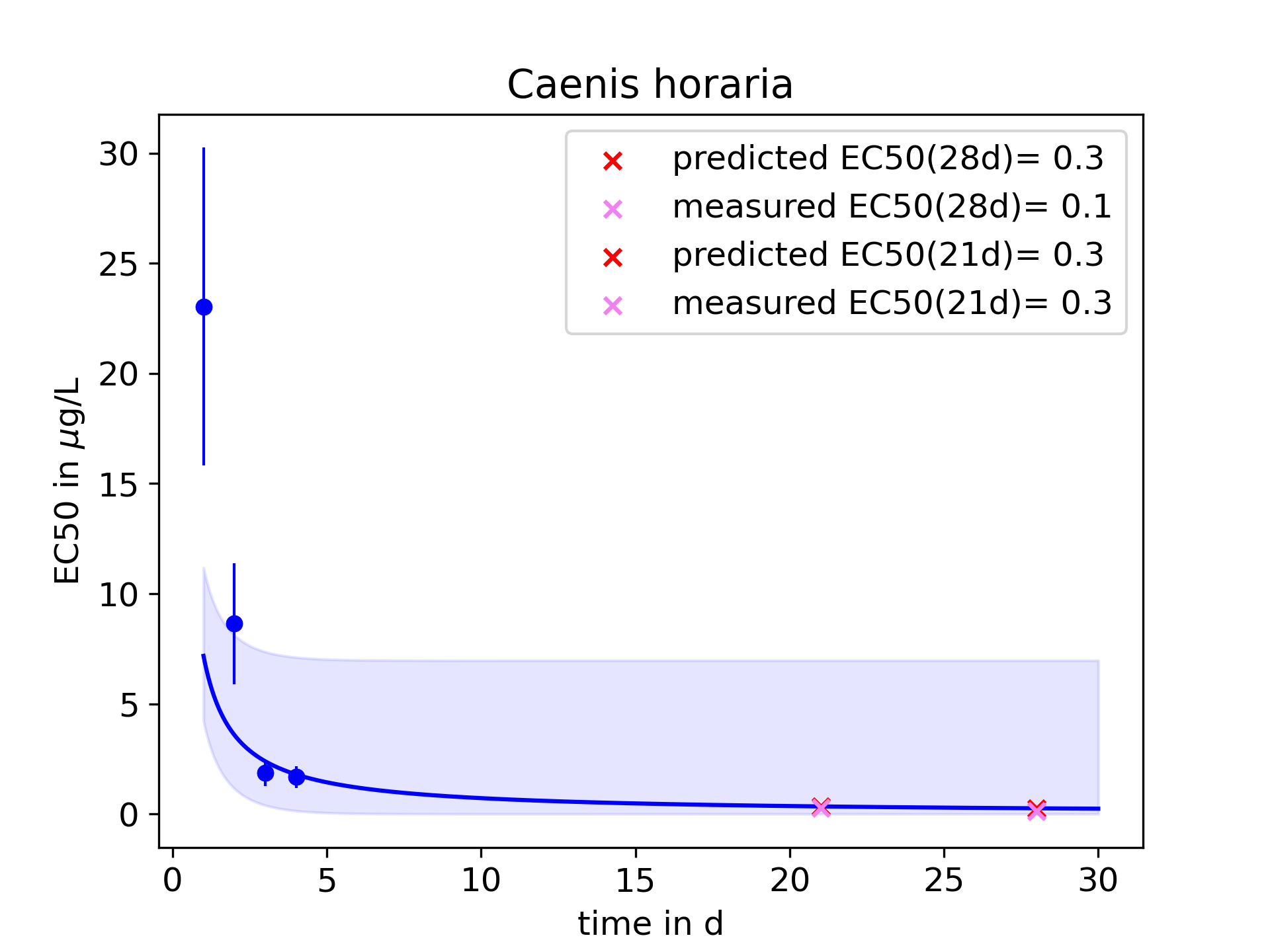

Supplement: vgaf015_Supplementary_Data [file vgaf015_supplementary_data.zip › vgaf015_Supplementary_Data/Figure A2b Caenis horaria EC50fit.tif]

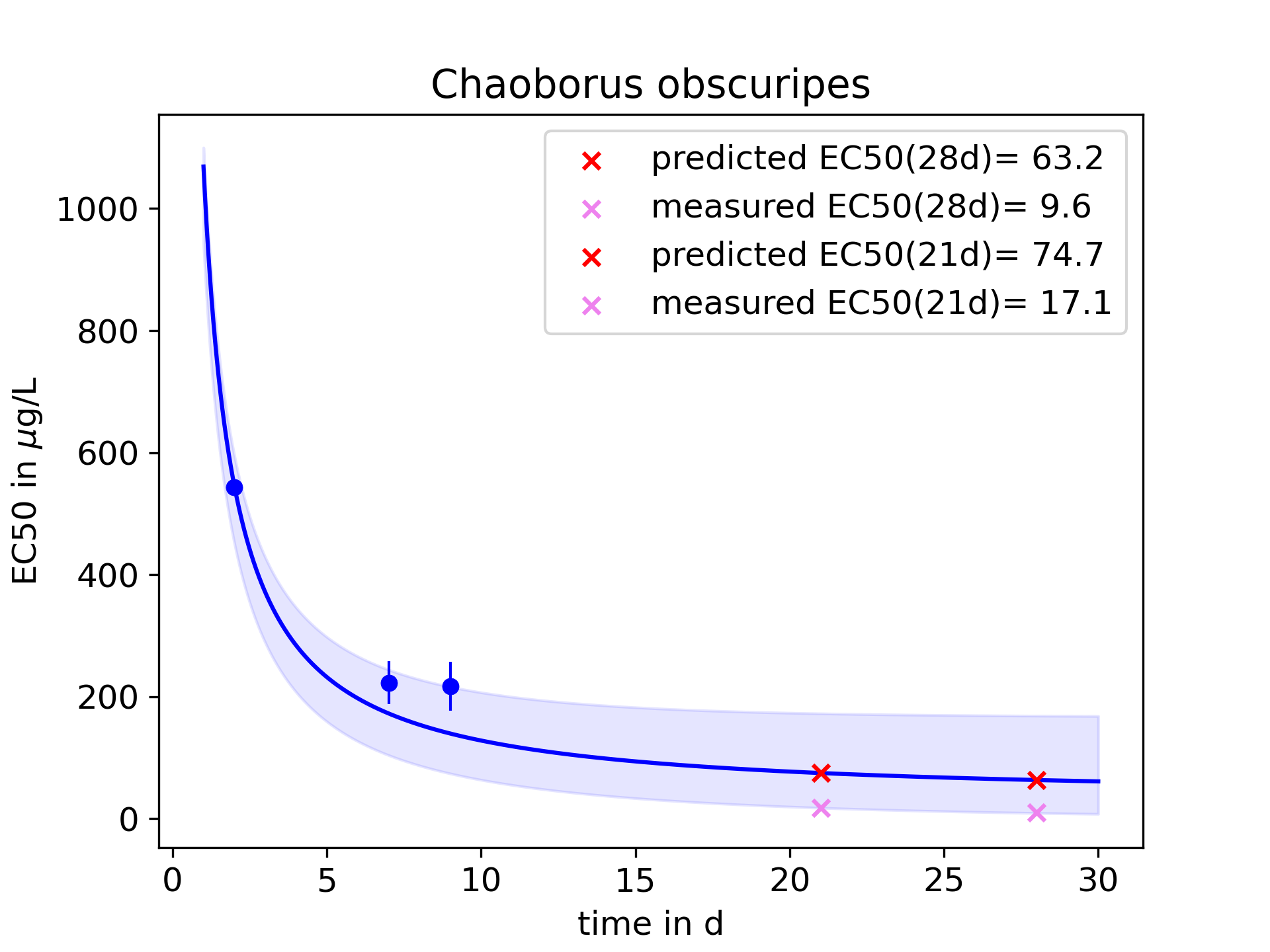

Supplement: vgaf015_Supplementary_Data [file vgaf015_supplementary_data.zip › vgaf015_Supplementary_Data/Figure A2c Chaoborus obscuripes EC50fit.tif]

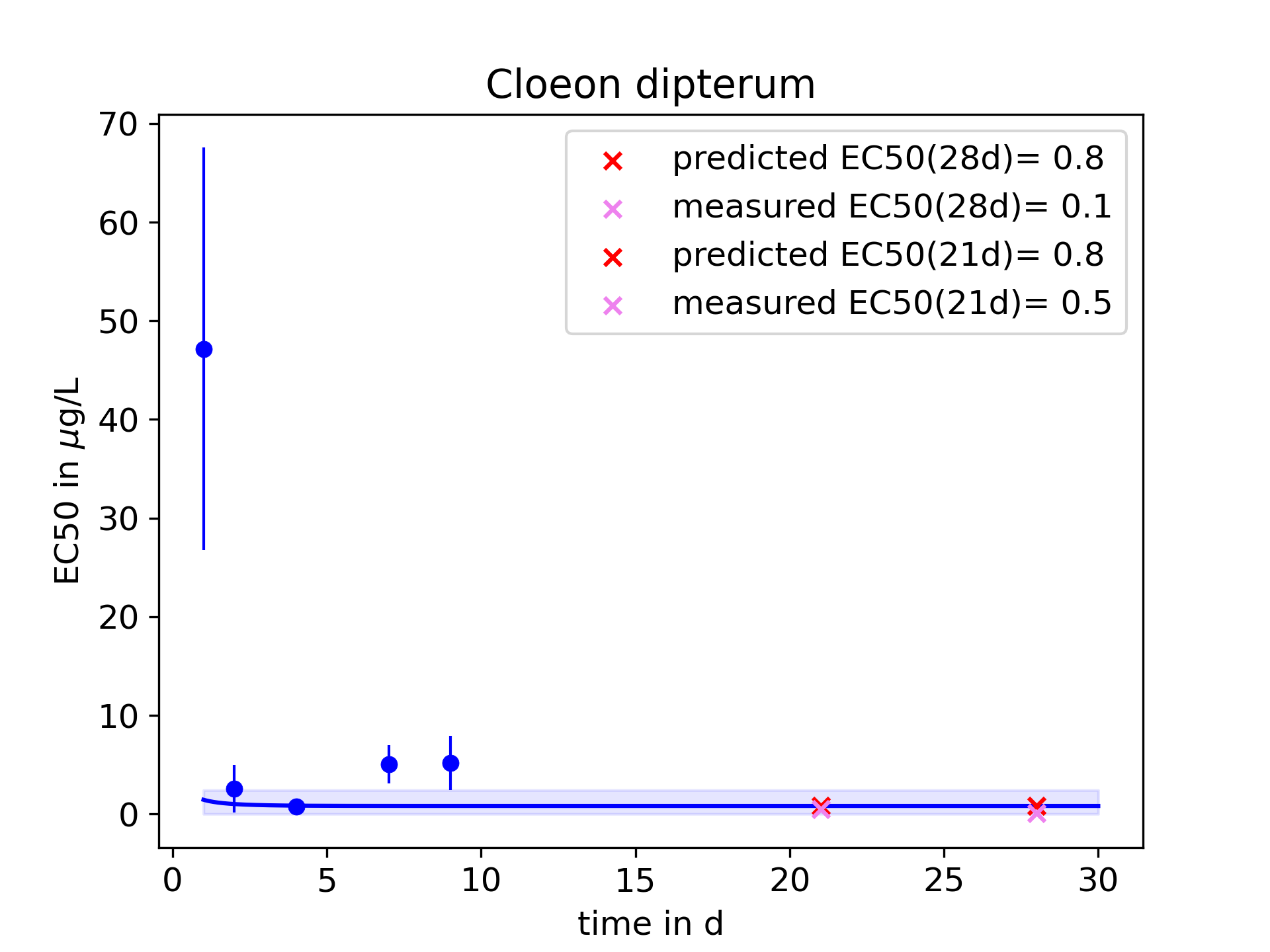

Supplement: vgaf015_Supplementary_Data [file vgaf015_supplementary_data.zip › vgaf015_Supplementary_Data/Figure A2d Cloeon dipterum EC50fit.tif]

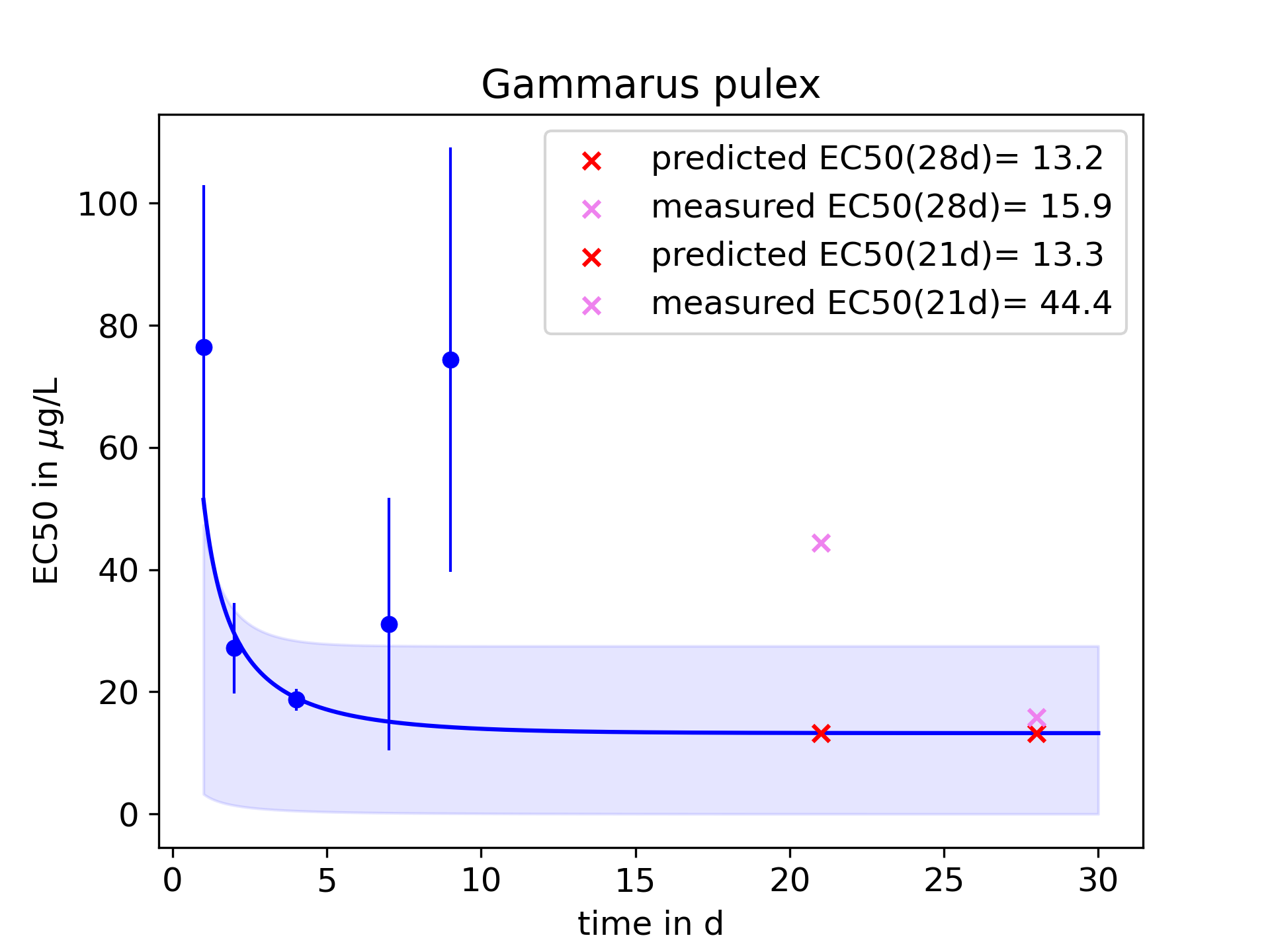

Supplement: vgaf015_Supplementary_Data [file vgaf015_supplementary_data.zip › vgaf015_Supplementary_Data/Figure A2e Gammarus pulex EC50fit.tif]

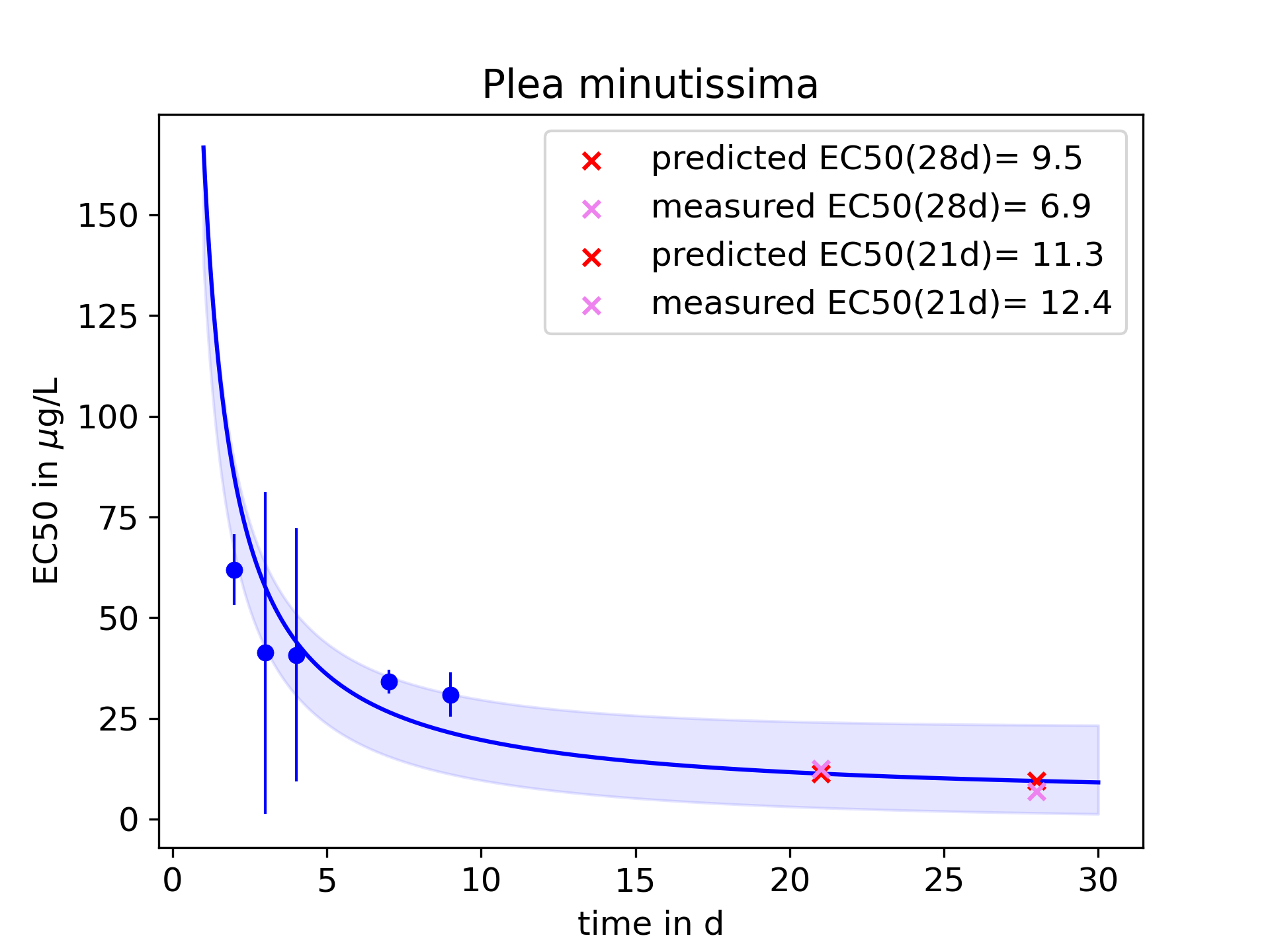

Supplement: vgaf015_Supplementary_Data [file vgaf015_supplementary_data.zip › vgaf015_Supplementary_Data/Figure A2f Plea minutissima EC50fit.tif]

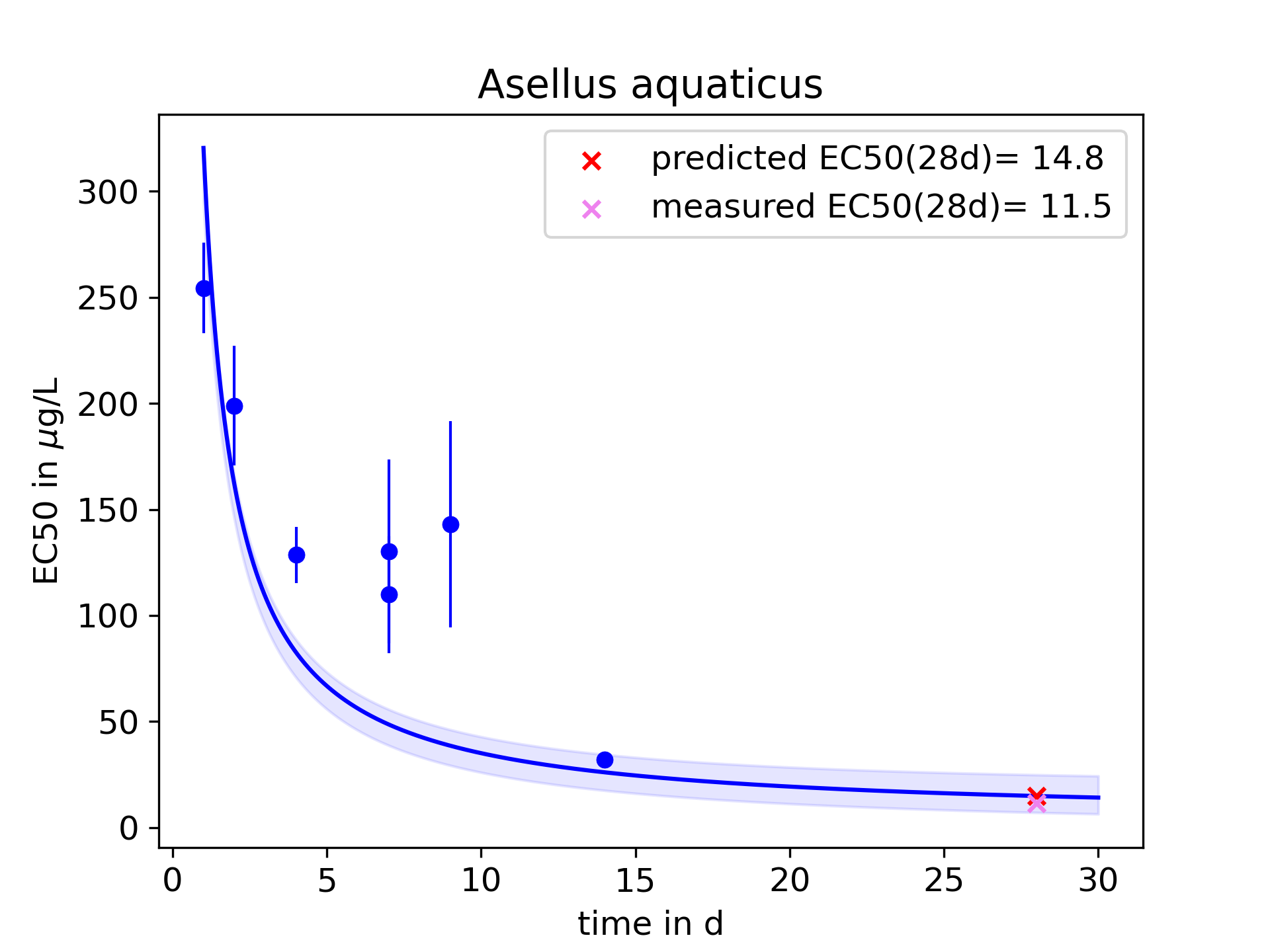

Supplement: vgaf015_Supplementary_Data [file vgaf015_supplementary_data.zip › vgaf015_Supplementary_Data/Figure A3a Asellus aquaticus EC50fit_with_chronic.tif]

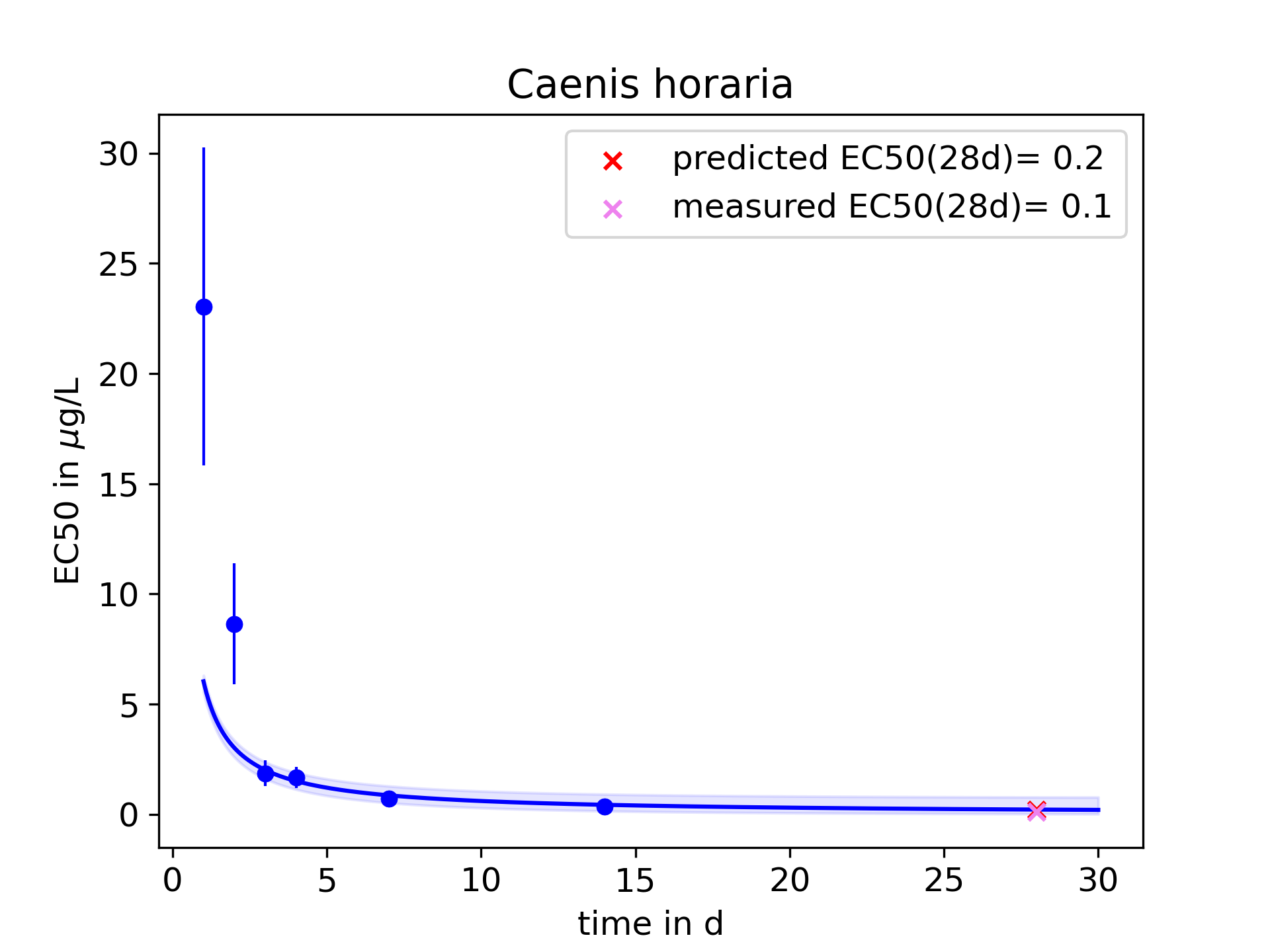

Supplement: vgaf015_Supplementary_Data [file vgaf015_supplementary_data.zip › vgaf015_Supplementary_Data/Figure A3b Caenis horaria EC50fit_with_chronic.tif]

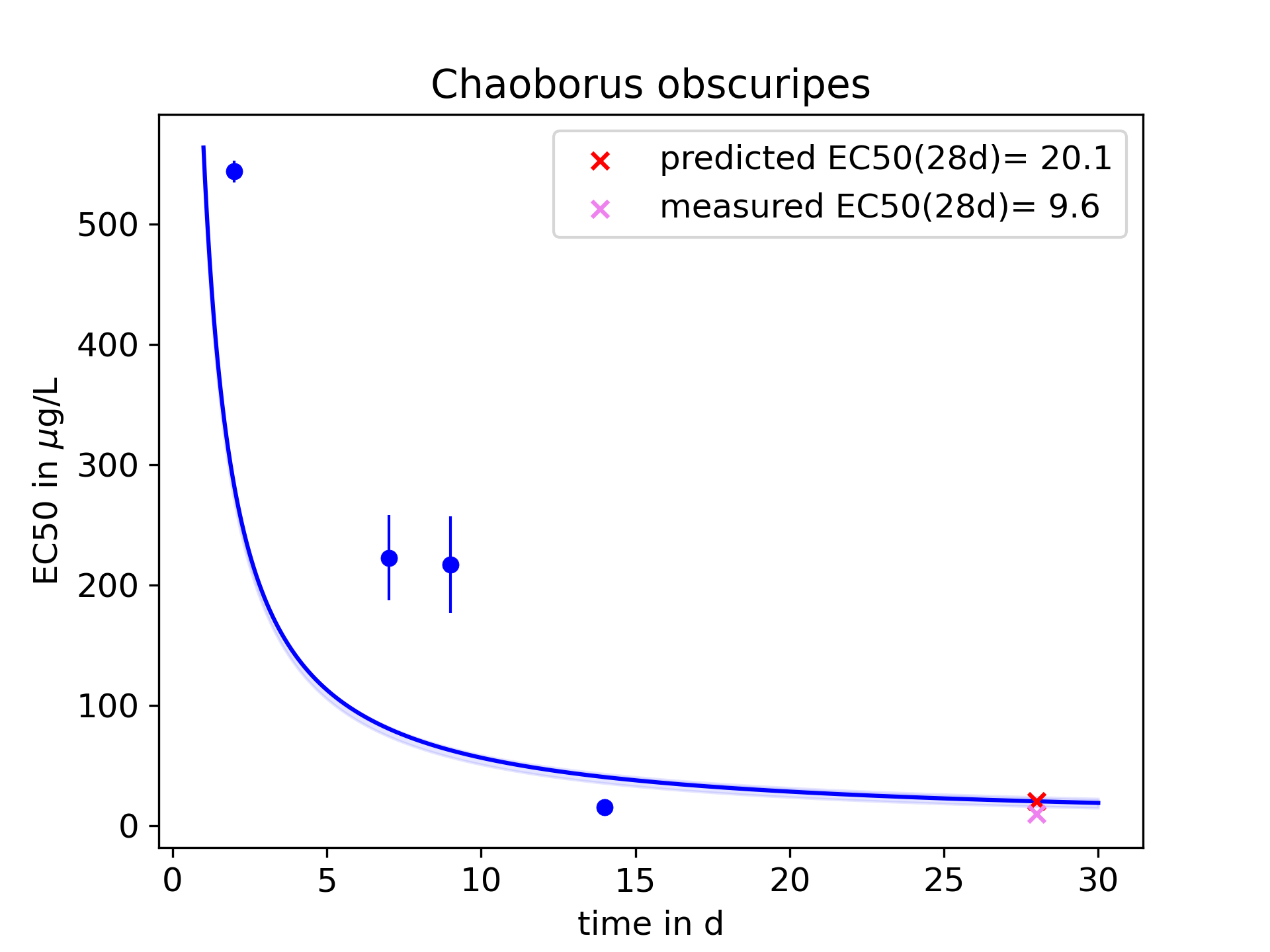

Supplement: vgaf015_Supplementary_Data [file vgaf015_supplementary_data.zip › vgaf015_Supplementary_Data/Figure A3c Chaoborus obscuripes EC50fit_with_chronic.tif]

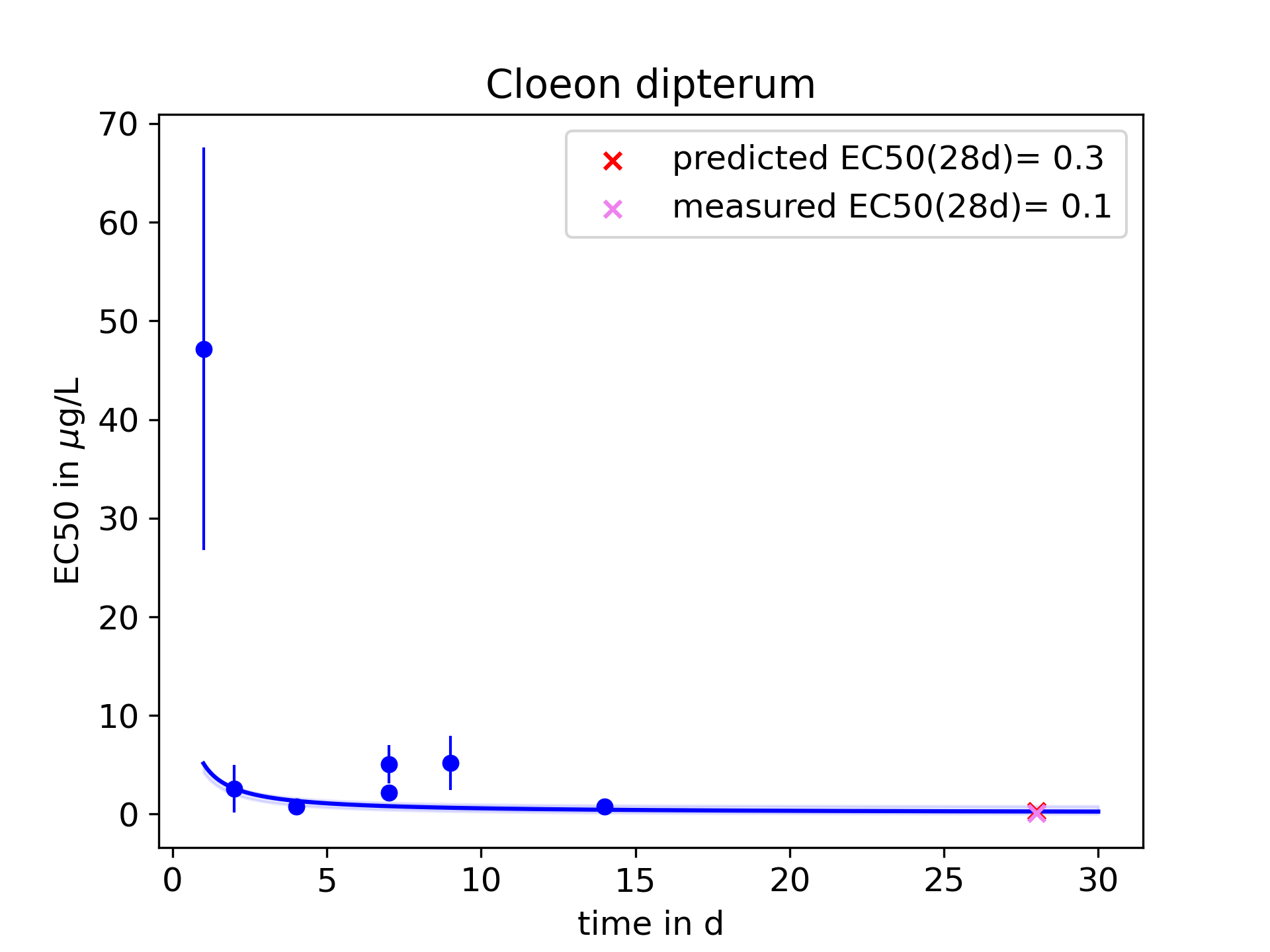

Supplement: vgaf015_Supplementary_Data [file vgaf015_supplementary_data.zip › vgaf015_Supplementary_Data/Figure A3d Cloeon dipterum EC50fit_with_chronic.tif]

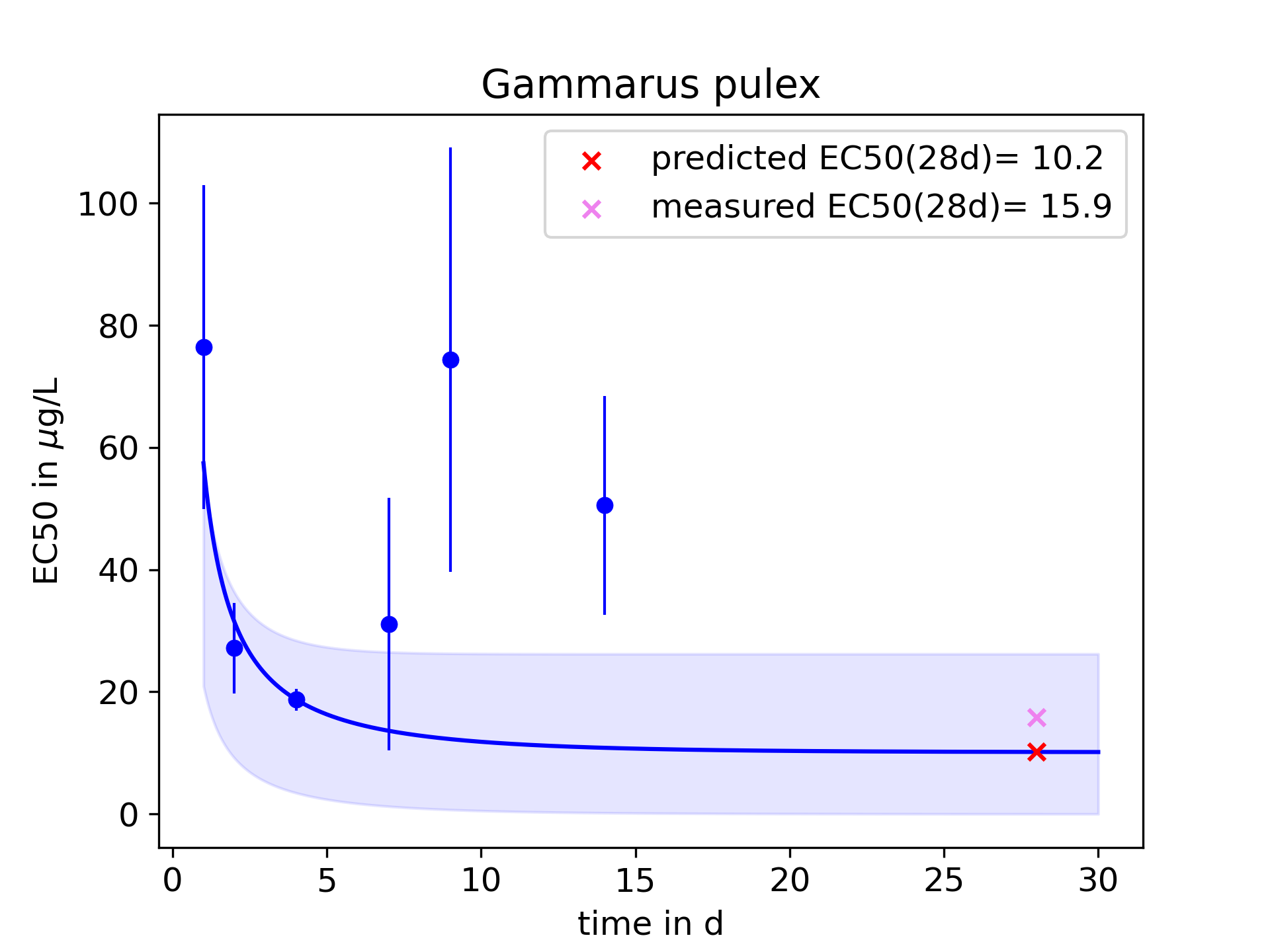

Supplement: vgaf015_Supplementary_Data [file vgaf015_supplementary_data.zip › vgaf015_Supplementary_Data/Figure A3e Gammarus pulex EC50fit_with_chronic.tif]

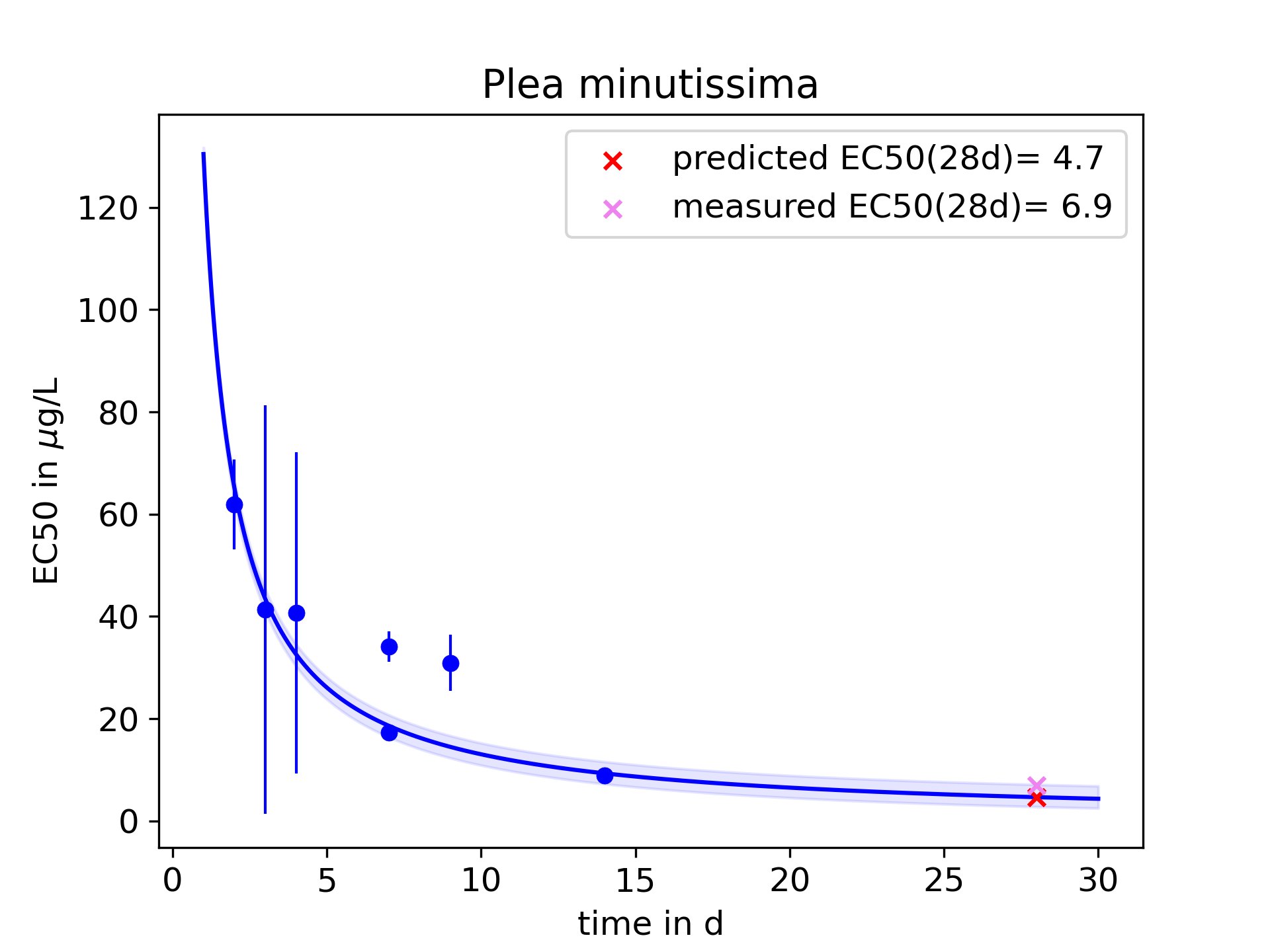

Supplement: vgaf015_Supplementary_Data [file vgaf015_supplementary_data.zip › vgaf015_Supplementary_Data/Figure A3f Plea minutissima EC50fit_with_chronic.tif]

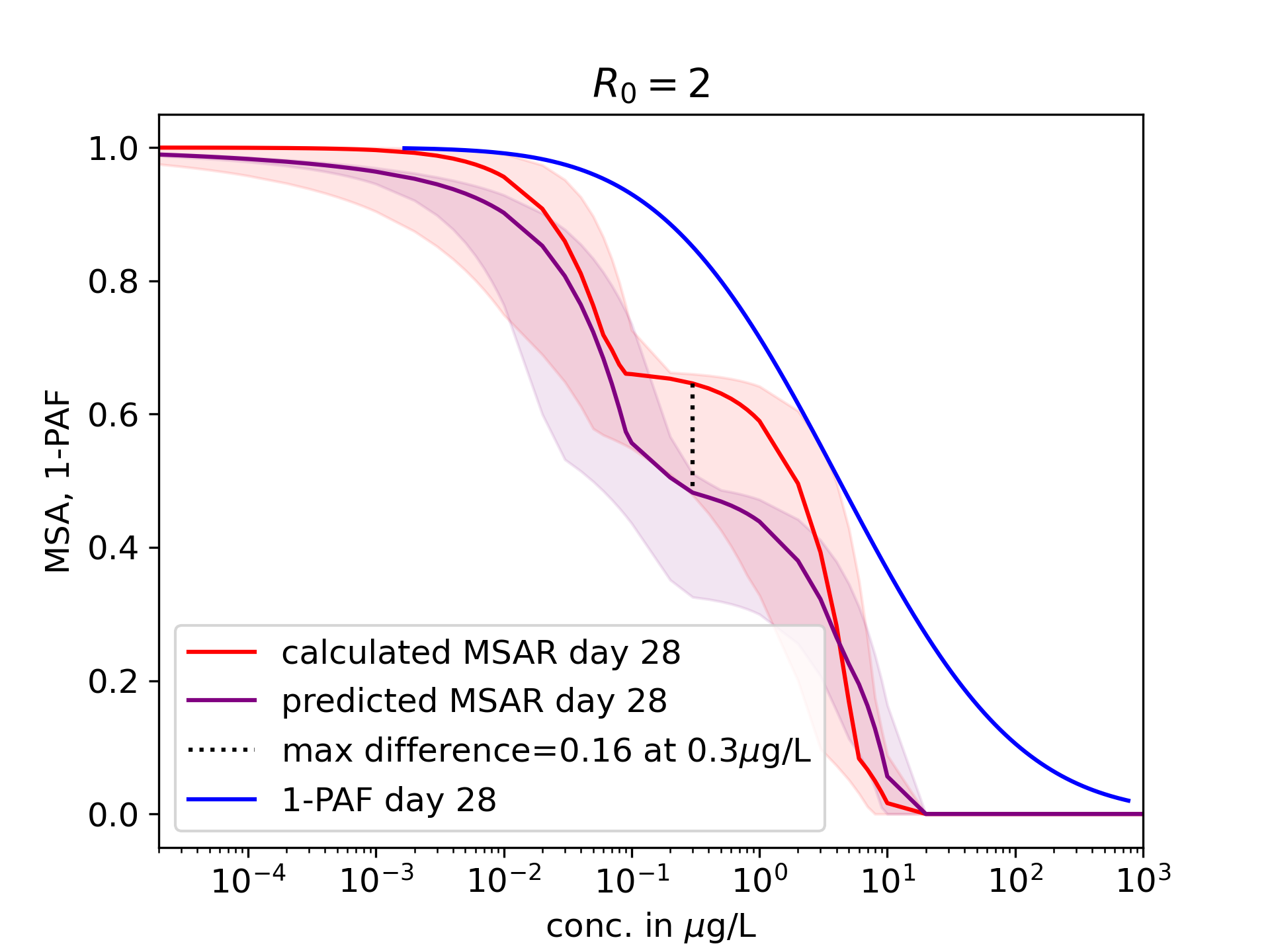

Supplement: vgaf015_Supplementary_Data [file vgaf015_supplementary_data.zip › vgaf015_Supplementary_Data/Figure A4a MSAR_with_chronic_28_R0_2.tif]

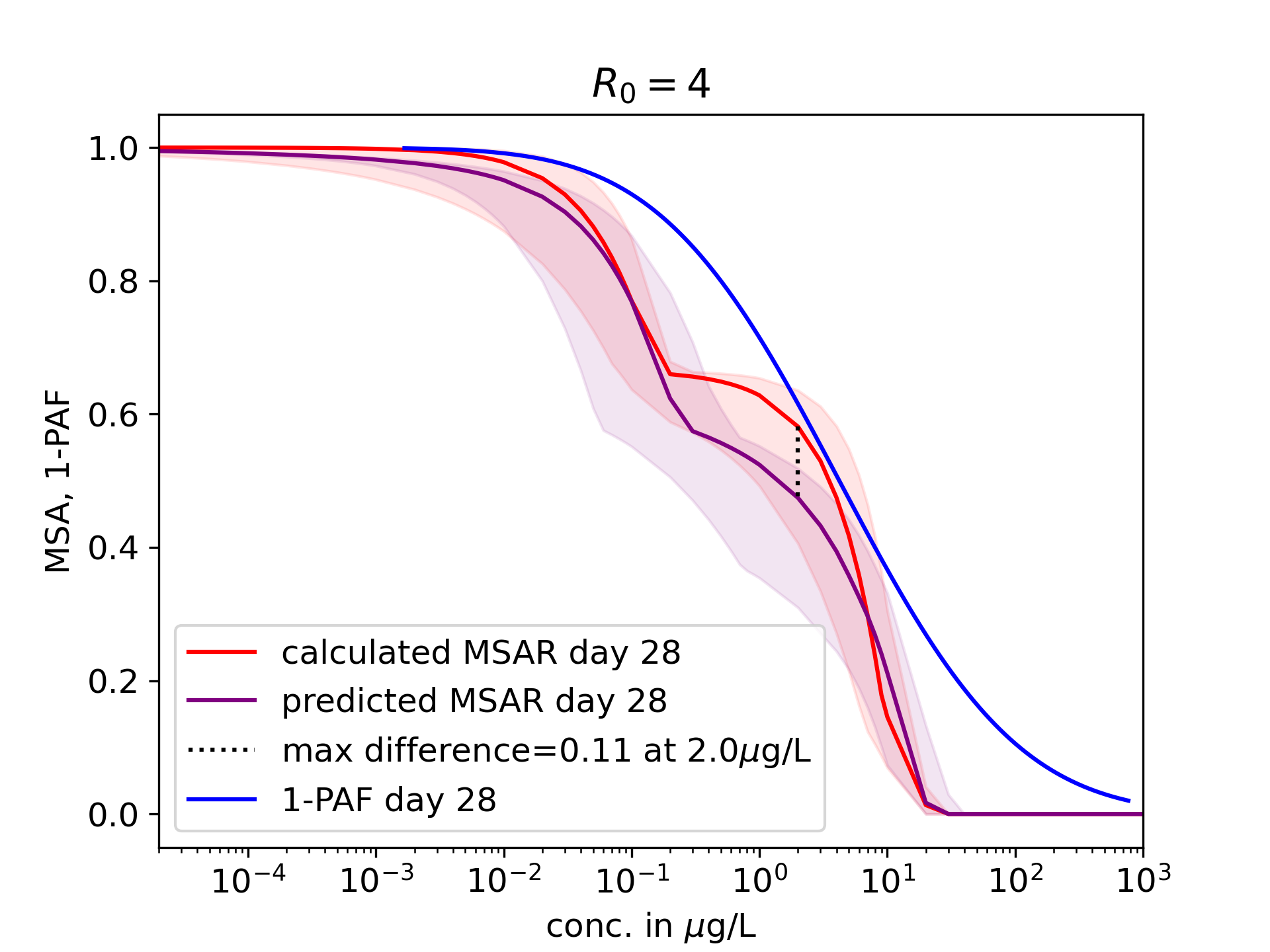

Supplement: vgaf015_Supplementary_Data [file vgaf015_supplementary_data.zip › vgaf015_Supplementary_Data/Figure A4b MSAR_with_chronic_28_R0_4.tif]

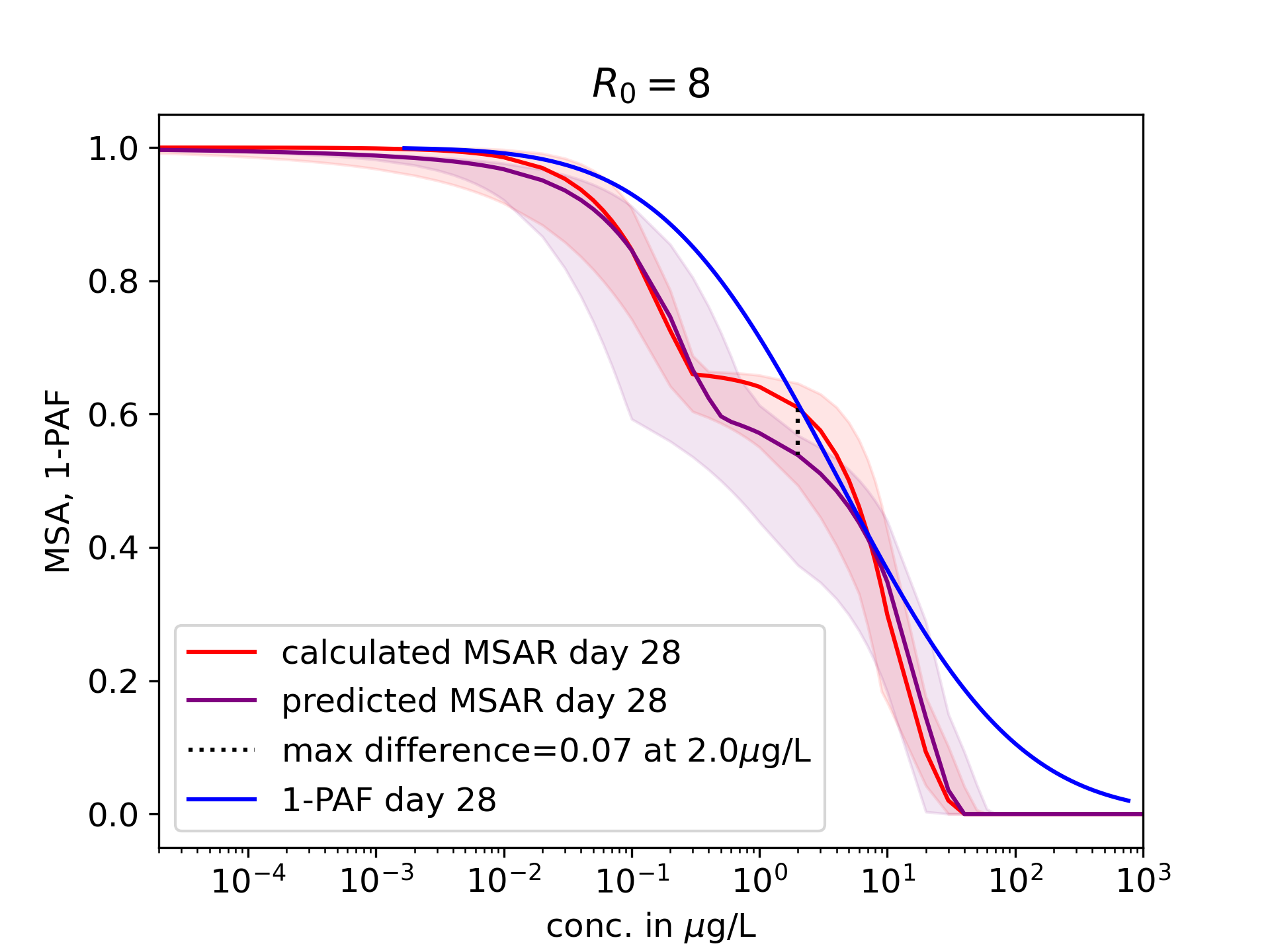

Supplement: vgaf015_Supplementary_Data [file vgaf015_supplementary_data.zip › vgaf015_Supplementary_Data/Figure A4c MSAR_with_chronic_28_R0_8.tif]

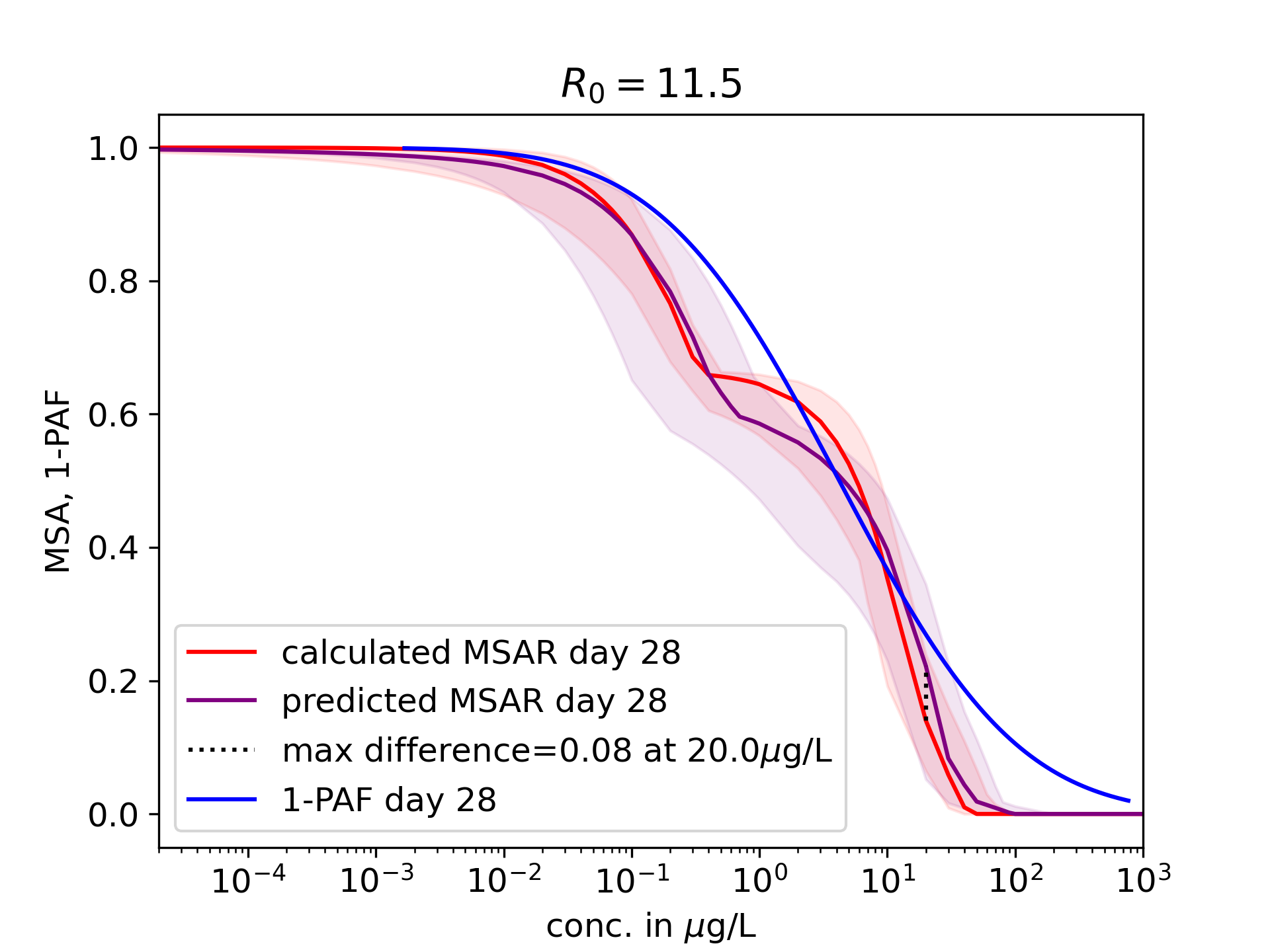

Supplement: vgaf015_Supplementary_Data [file vgaf015_supplementary_data.zip › vgaf015_Supplementary_Data/Figure A4d MSAR_with_chronic_28_R0_11.5.tif]
